# Zero-inflated and distributed lag nonlinear models with random effects for assessing environmental impacts on respiratory health in peripheral regions of Costa Rica

**DOI:** 10.3389/fpubh.2026.1753511

**Published:** 2026-04-08

**Authors:** Shu Wei Chou-Chen, Emanuelle Parra-Rodríguez

**Affiliations:** 1 School of Statistics and Research Center in Pure and Applied Mathematics, University of Costa Rica, San José, Costa Rica; 2 School of Mathematics, Costa Rica Institute of Technology, Cartago, Costa Rica; 3 School of Mathematics, University of Costa Rica, San José, Costa Rica

**Keywords:** distributed lag nonlinear model (DLNM), hospital discharges, mixed model, random effects, respiratory health, zero inflation

## Abstract

**Introduction:**

Climate change and air pollution are key determinants of public health, particularly in the onset and exacerbation of respiratory diseases. The main objective is to quantify the lagged and nonlinear effects of climate and air pollution on respiratory hospitalizations in peripheral regions of Costa Rica.

**Methods:**

This study presents a methodological framework that combines Distributed Lag Nonlinear Models (DLNM) with Generalized Linear Mixed Models (GLMM), incorporating fixed and random effects, to assess the lagged and nonlinear effects of climatic variables and atmospheric pollutants on hospitalizations due to respiratory causes. The response specification was carried out using zero-inflated distributions, aiming to adequately capture the overdispersion and excess zeros present in the data. The analysis focused on peripheral climatological regions and subregions of Costa Rica-territories outside the Central Valley, including Caribbean and Pacific coasts and border areas, characterized by low population density. Weekly data (2000–2019) on temperature, precipitation, relative humidity, and aerosol optical depth (AOD) were combined with seasonal effects and a population offset to account for subregional differences.

**Results:**

Northern and Central Pacific regions show similar climate–pollution impacts on respiratory health, while the South Pacific exhibits stronger and more persistent risks from moderate to high pollution, and Atlantic regions show consistently higher risks associated with intense rainfall and high humidity. Overall, precipitation extremes, high humidity, and AOD contribute more to respiratory hospitalizations than temperature.

**Conclusion:**

This approach improves explanatory and predictive performance, yields robust relative risk estimates, and captures regional sensitivity to environmental conditions, supporting spatiotemporal health analysis and early warning systems in rural tropical settings.

## 1 Introduction

Respiratory health is a crucial component of public health, encompassing a range of conditions such as asthma, chronic obstructive pulmonary disease (COPD), and acute respiratory infections. These disorders contribute substantially to global morbidity and mortality, placing significant pressure on healthcare systems (1).

Climate variability and air pollution are major environmental determinants of respiratory health (2). Climate change increases the frequency of extreme events such as floods and other natural disasters, while extremes of temperature, humidity, and seasonal fluctuations affect the transmission and severity of respiratory diseases (3). In addition, pollutants such as particulate matter, ozone, and nitrogen dioxide exacerbate symptoms and raise hospitalization rates. Collectively, these factors intensify healthcare burdens and pose escalating challenges for medical professional and policymakers worldwide (4–8).

Although several studies have documented these associations, methodological challenges remain in accurately capturing the complex nonlinear and lagged effects on respiratory health data. Temperature has been reported to have a nonlinear effect on respiratory outcomes, typically characterized by a U-shaped association. In other words, both extremely low and high temperatures are associated with increased risks of hospitalization (9–11). However, other studies have found that only extreme high temperatures significantly affect respiratory and cardiovascular diseases (12).

Other climatic exposures, such as precipitation and relative humidity, have also been linked to respiratory outcomes. For instance, Pedder et al. (10) reported that only low relative humidity (RH) was associated with increased pneumonia hospital admissions, whereas Tran et al. (11) observed that both extremely high and low RH were associated with respiratory hospitalizations. Similarly, higher precipitation levels have been found to correlate with an increase in respiratory and cardiovascular hospital admissions (13). Finally, air pollution–particularly particulate matter (*PM*_10_)–has been consistently reported to have a positive association with adverse respiratory health outcomes (13, 14). This variability in findings is further amplified by climatic fluctuations and geographically diverse contexts characterized by population and socioeconomic heterogeneity.

This study faces these challenges and seeks to address them through assessing the nonlinear and lagged associations between climatic variables (temperature, precipitation, and relative humidity), air pollution (measured by aerosol optical depth, AOD), and hospital discharges due to respiratory causes in Costa Rica, a Central American country with tropical and sub-tropical climates and a wide range of microclimates. Hospital discharge data are used because they provide accurate diagnoses of respiratory conditions within the country's health administrative system. A previous study applied several predictive machine learning methods to forecast respiratory hospitalizations based on climatic and pollution data (15).

Moreover, the analysis focuses on peripheral climatological regions of Costa Rica, including the Caribbean and Pacific coasts and border areas, which are characterized by lower population density and pronounced climatic variability (16). The Central Valley is excluded due to its large population, high incidence of respiratory hospitalizations, and the need for a distinct modeling approach.

To characterize the dynamics between respiratory hospitalizations and their potential nonlinear and lagged climatic effects, we proposed the distributed lag nonlinear models (DLNMs) (17–19) with random effects to account subregional variability, focusing on weekly respiratory count data. Recent methodological developments have extended DLNMs to hierarchical and mixed-effects frameworks for multi-location environmental time-series analyses. These approaches allow simultaneous estimation of region-specific exposure-lag-response associations while accounting for between-area heterogeneity (20). Large-scale applications have combined DLNM structures with hierarchical meta-regression models to quantify spatial variability in temperature-related health risks (21). Contemporary respiratory health studies continue to apply DLNM-based specifications under diverse climatic regimes (22), supporting their integration with random-effects structures in geographically stratified epidemiological settings.

In peripheral tropical regions characterized by lower population density and pronounced climatic variability, weekly aggregated health counts frequently exhibit overdispersion and excess zeros due to sparse population structures or episodic disease occurrence (11, 23). To accommodate these low-count features, we incorporated zero-inflated negative binomial distributions, which allow the separation of structural absence of events from stochastic variability while addressing overdispersion (24, 25). Model estimation was implemented within flexible mixed-effects frameworks that support hierarchical structures and computational stability in complex regression settings (26–28). By jointly modeling nonlinear lagged exposure effects and inter-subregional heterogeneity within this framework, the proposed approach provides robust quantitative evidence of the influence of climatic and air-quality variables on respiratory hospital discharges.

From the selected models, Northern and Central Pacific regions exhibit broadly similar climate–pollution patterns in their impacts on respiratory health, reflecting shared climatic dynamics. In contrast, the South Pacific region demonstrates a stronger and more persistent increase in respiratory risk associated with moderate to high pollution levels. Atlantic regions, despite their heterogeneous microclimatic conditions, consistently show elevated respiratory risks linked to intense precipitation, high relative humidity, and cooler microclimates. In summary, extreme precipitation, elevated humidity, and increased aerosol loads play a more substantial role in driving respiratory hospitalizations than temperature, while satellite-derived aerosol optical depth likely underestimates surface-level pollution exposure in Pacific regions during dry and atmospherically stable conditions.

Overall, this study demonstrates a consistent methodological framework that provides quantitative evidence of the nonlinear lagged influence of climate variables on respiratory discharges, while incorporating random effects to account for inter-subregional heterogeneity in small-population settings. This evidence supports epidemiological surveillance and the development of early warning thresholds in tropical peripheral regions with differential risks, consistent with recent work in non-temperate climates (10, 11, 29) and previous applications of DLNM (30).

## 2 Materials and methods

### 2.1 Study area

**Figure 1:**
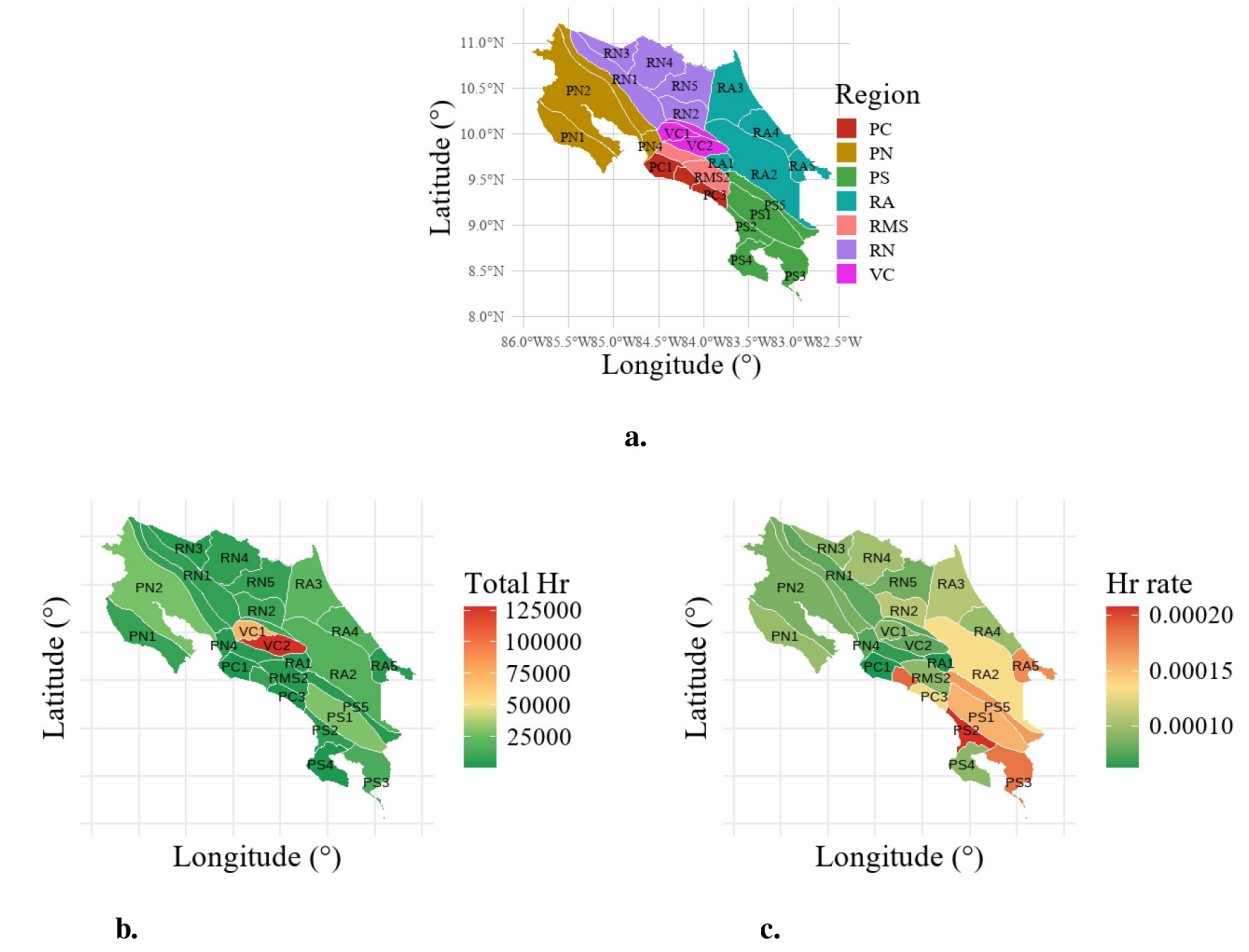
Climatic regions, subregions, and hospital discharges (Hr) and Hr rate per subregion in Costa Rica. **(a)** Climatic regions and subregions. **(b)** Hospital discharge by subregion. **(c)** Hospital discharge rate by subregion.

This study focuses on Costa Rica, a country where diverse climatic conditions strongly influence the incidence of respiratory diseases (31). Illnesses such as asthma, bronchitis, and pneumonia are major causes of morbidity and hospitalization, particularly among children and the older population (32). The tropical and subtropical climate, characterized by high humidity and variable temperatures, plays a central role in both the prevalence and exacerbation of these conditions (6, 31, 33). According to the National Meteorological Institute (16), Costa Rica is divided into seven main climatic regions and several subregions, each defined by distinct patterns of temperature, precipitation, and dry season duration. Table 1 summarizes the principal characteristics of each climatic region and Figure 1a shows the map of the study area. VC is excluded from the study due to its high population density and large number of hospital discharges, which require a different modeling approach (see Figure 1b).

**Table 1:** Description of climatic and subclimatic regions of Costa Rica.

Climate region	Description	Subregions
North Pacific Region (PN)	Temperate and tropical climates with a dry season, influenced by geographic factors.	Western Nicoya Peninsula (PN1), Central North Pacific (PN2), foothills of the Guanacaste and Tilarán ranges (PN3), and the lower basins of the Barranca and Grande de Tárcoles rivers (PN4).
Central Pacific Region (PC)	Tropical climate with a marked rainy season and a short, moderate dry season.	Parrita Valley (PC1), Naranjo River Basin, Quepos (PC2), and Barú River Basin, Dominical (PC3).
South Pacific Region (PS)	Unique rainfall regime with a very short dry period and intense rainfall throughout the year.	Valle del General and Coto Brus (PS1), Diquís Valley (PS2), Coto Colorado Valley (PS3), Osa Peninsula (PS4), and the Pacific slopes of the Talamanca Range (PS5).
Southern Mountain Region (RMS)	Highland valleys that result in warm temperatures in lower areas and cold conditions in higher zones.	Upper Basins of the Turrubares and Tulín rivers, and the Candelaria Valley (RMS1), and Upper Basin of the Pirrís or Parrita River (RMS2).
Central Valley Region (VC)	This region is influenced by both Caribbean and Pacific foothill climates, with areas where the dry season is reduced to one month and pockets of temperate climate.	Western Central Valley (VC1), Eastern Central Valley (VC2), and the slopes of the Central Volcanic Range (Poás, Barva, and Irazú) (VC3). This region has the highest population density and urbanization levels and is also among the most exposed to environmental pollutants.
Northern Region (RN)	It belongs to the Caribbean precipitation regime, which is rainy year-round, with relatively drier months in February, March, and October.	Eastern slopes of the Guanacaste and Tilarán ranges (RN1), Northern slopes of the Central Volcanic Range (RN2), Guatusos Plains (RN3), San Carlos Plains (RN4), and Sarapiquí Plains (RN5).
Atlantic Region (RA)	This humid tropical region experiences abundant rainfall, especially in the mountainous areas where it rains year-round.	Basins of the Macho, Grande de Orosí, and Pejibaye rivers (RA1), Atlantic slopes of the Irazú-Turrialba Massif and the Talamanca Range – Reventazón Valley (RA2), Tortuguero Plains (RA3), Santa Clara and Matina Plains and the Banano River Basin (RA4), and the southern area of the Banano River Basin (RA5).

### 2.2 Data

Daily hospital discharge records due to respiratory causes [ICD-10 codes J00-J99 (34)], provided by the Costa Rican Social Security Fund (CCSS), were aggregated by epidemiological week to ensure consistency with the temporal resolution of environmental exposures. The dependent variable therefore corresponds to the weekly count of respiratory hospital discharges per climatic subregion from 2000 to 2019 across 27 peripheral climatological subregions of Costa Rica (16). The Central Valley is excluded because of its large population, high rates of respiratory hospitalizations, and a separate modeling strategy is required (Figures 1b, c). See also Supplementary Figure S1 for time series plots.

Climatic exposures were obtained using satellite-derived data on mean temperature (T) (35), precipitation (P) (36), relative humidity (RH) (37), and aerosol optical depth (AOD) (38), as a proxy for air pollution (39, 40). Both AOD and relative humidity data contained missing values across several subregions. To address missing values in aerosol optical depth (AOD) and relative humidity (RH), we applied an inverse distance weighting (IDW) interpolation method (41, 42), using Euclidean distances between each satellite grid cell and the centroid of each climatic subregion. For a given week and subregion, exposure values were aggregated as a weighted average of surrounding grid cells according to


$$\begin{array}{l} {\hat{X}}_{it}=\frac{\sum_{j=1}^{m}w_{ij}X_{jt}}{\sum_{j=1}^{m}w_{ij}},\;w_{ij}=\frac{1}{d_{ij}^{p}}, \end{array}$$

where ${\hat{X}}_{it}$ denotes the interpolated exposure (AOD or RH) for subregion *i* at week *t*, *X*_*jt*_ is the observed exposure at grid cell *j*, *d*_*ij*_ is the Euclidean distance between the centroid of subregion *i* and grid cell *j*, and *p* is the distance decay parameter. In this study, we set *p* = 1, assigning greater influence to nearby grid cells while preserving smooth spatial transitions.

Following an exploratory analysis, a marked spatial heterogeneity was observed both in the magnitude and variability of respiratory hospital discharges, as well as in climatic conditions. Although the overall proportion of zero counts in the full dataset was 5.44%, these zeros were primarily concentrated in subregions with low population density, supporting the use of count models capable of handling zero inflation (For further details, see the selected summary statistics in Table 2.

**Table 2:** Descriptive statistics (mean, first-third quantiles, maximum and percentage of zeros) for weekly hospital discharges (*Y*) and weekly mean climatic covariates–temperature (*T*), precipitation (*P*), relative humidity (*RH*), and aerosol optical depth (*AOD*)–by climatic subregion during 2000–2019.

Subregion	Hospital discharge (*Y*)						*P*	*T*	*RH*	*AOD*
	Mean	*Q*1	*Median*	*Q*3	*Max*	Zeros (%)				
PC1	1.06	0	1	2	5	35.96	9.61	29.3	0.372	1.94 × 10^−4^
PC2	7.67	5	7	10	22	0.10	12.7	29.8	0.370	1.92 × 10^−4^
PC3	0.763	0	1	1	5	48.56	14.4	27.6	0.369	1.91 × 10^−4^
PN1	8.02	6	8	10	23	0.19	7.92	29.8	0.375	1.97 × 10^−4^
PN2	27.4	21	27	32.5	62	0.00	6.96	30.0	0.376	1.96 × 10^−4^
PN3	7.03	5	6	9	20	0.19	8.26	26.9	0.375	1.94 × 10^−4^
PN4	3.79	2	4	5	14	3.65	7.75	29.0	0.373	1.93 × 10^−4^
PS1	28.7	22	27	34	63	0.00	12.0	23.5	0.368	1.88 × 10^−4^
PS2	5.38	3	5	7	20	0.87	11.3	27.9	0.367	1.90 × 10^−4^
PS3	14.7	11	14	18	45	0.00	14.9	28.4	0.367	1.89 × 10^−4^
PS4	1.25	0	1	2	7	32.31	14.3	30.1	0.366	1.91 × 10^−4^
PS5	3.89	2	4	5	17	3.56	12.6	20.7	0.368	1.87 × 10^−4^
RA1	3.28	2	3	4	13	5.87	12.4	18.7	0.370	1.89 × 10^−4^
RA2	18.1	14	17	22	49	0.00	15.2	21.6	0.370	1.86 × 10^−4^
RA3	21.4	17	21	25	48	0.00	18.2	28.4	0.372	1.86 × 10^−4^
RA4	17.0	13	16	21	40	0.10	18.7	27.6	0.371	1.84 × 10^−4^
RA5	4.33	2	4	6	20	3.65	15.0	28.8	0.369	1.84 × 10^−4^
RMS1	4.33	3	4	6	12	1.83	11.1	24.9	0.372	1.92 × 10^−4^
RMS2	3.88	2	3	5	18	3.75	12.8	21.5	0.370	1.90 × 10^−4^
RN1	6.92	5	7	9	20	0.19	11.7	27.2	0.375	1.93 × 10^−4^
RN2	10.1	7	10	12	30	0.00	13.6	23.4	0.373	1.90 × 10^−4^
RN3	3.78	2	3	5	15	5.00	9.81	30.9	0.375	1.92 × 10^−4^
RN4	6.14	4	6	8	21	1.06	13.0	29.5	0.375	1.91 × 10^−4^
RN5	7.33	5	7	9	23	0.38	16.1	28.6	0.374	1.89 × 10^−4^

### 2.3 Model

Each region exhibits unique climatic conditions with only slight variation across its subregions; therefore, our aim is to capture differences in nonlinear and lagged associations across climatic regions. For this end, we propose a distributed lag nonlinear model (DLNM) with a random intercept for subregions to model weekly hospital discharges for each region, using Poisson (PO), negative binomial (NegBin) distributions, and their zero-inflated counterparts (ZIP and ZINBI, respectively) to account for the excess zeros.

For a given climate region, the hospital discharges *y*_*it*_ in subregion *i* at week *t* are modeled using a count distribution. Specifically, *Y*_*it*_~Poisson(μ_*it*_) or *Y*_*it*_~NegBin(μ_*it*_, κ) with mean μ_*it*_:


$$\begin{array}{l} \log\left(\mu_{it}\right)=\beta_{0}+\overset{4}{\underset{v=1}{\sum }}s_{v}(X_{vit},l_{v})+\gamma_{s}^{\prime }D_{t}+b_{i}+\log\left(N_{it}\right), \end{array}$$

where β_0_ is the intercept, $b_{i}\sim N\left(0,\sigma_{b}^{2}\right)$ is the random intercept capturing subregional variability, *D*_*t*_ is a vector of 51 dummy variables that account for the seasonal effect with 52 levels, and γ denotes the corresponding coefficients, and log(*N*_*it*_) is the offset accounting the population at risk. Additionally, *s*_*v*_(·) is *cross-basis* functions representing nonlinear exposure-response and lag associations for each exposure predictor *v*, of the form


$$\begin{array}{l} s_{v}\left(x_{t};\beta \right)=\overset{L}{\underset{l=0}{\sum }}\overset{v_{x}}{\underset{j=1}{\sum }}\overset{v_{l}}{\underset{k=1}{\sum }}b_{j}(x_{t-l})h_{k}\left(l\right)\beta_{jk}, \end{array}$$

with *x*_*t*_ denoting exposure at time *t*; *L* the maximum lag (with *l* = 0, 1, …, *L*); *v*_*x*_ and *v*_*l*_ the degrees of freedom for spline basis functions in the exposure and lag spaces, respectively; *b*_*j*_(·) the *j*th basis in exposure evaluated at *x*_*t*−*l*_; and *h*_*k*_(·) the *k*th basis function evaluated at *l* (17, 18, 43, 44).

Overdispersion was assessed in the Poisson models by examining the ratio of residual deviance to degrees of freedom. In all climatic regions, this ratio exceeded 1, indicating extra-Poisson variability (45, 46). Consequently, negative binomial models were considered, as they include an additional dispersion parameter allowing the variance to exceed the mean. Model selection was further supported by information criteria (AIC, BIC) and predictive accuracy measures (MAE, RMSE), ensuring both goodness-of-fit and parsimony.

In the presence of excess zeros, we extend the count family to zero-inflated specifications with density function:


$$f_{Y}=\pi_{it}\delta_{0}+\left(1-\pi_{it}\right)f_{Y}\left(\mu_{it}\right),$$

where π_*it*_ (logit link) models structural zeros and *f*_*Y*_*it*__(·) is either Poisson or negative binomial (27, 28, 47). In the zero-inflated specifications, the inflation probability is modeled using a logit link with an intercept-only structure, while all climatic exposures, cross-basis terms, seasonal indicators, offset, and subregional random effects are included exclusively in the count component. Thus, zero inflation captures structural excess zeros not explained by the covariates, without introducing additional predictors in the inflation equation. We refer them as DLNM-ZIP_*rf*_ and DLNM-ZINBI_*rf*_, respectively. This decouples zero generation from count dynamics–critical in sparsely populated subregions, especially peripheral or coastal areas of Costa Rica with low population density, limited hospital access, and higher frequency of incomplete records.

The specification of the DLNM requires determining the relevant lag structure and degree of nonlinearity for each climatic exposure. To this end, an exploratory dependence analysis between each exposure and the response variable was conducted for lags up to 14 weeks in each region and subregion. A maximum lag of 14 weeks was selected as an upper bound, consistent with epidemiological evidence indicating that delayed respiratory responses to climatic exposures may extend over multiple weeks, while avoiding unnecessarily long lag windows that could induce overfitting (15, 17, 18).

Pearson correlation (capturing linear dependence) and distance correlation and mutual information (capturing both linear and nonlinear dependence) were computed at each lag. Statistical significance of all measures was assessed using a non-parametric permutation test (44, 48–52), allowing explicit comparison between purely linear associations and more general dependence structures. The maximum lag was defined as the largest lag at which at least two of the three dependence measures remained statistically significant (*p* < 0.05), ensuring robustness under both linear and nonlinear dependence.

To capture nonlinear exposure-lag relationships, cubic spline bases were specified for both the predictor and lag spaces. Alternative specifications with 2 and 4 degrees of freedom were evaluated; however, 3 degrees of freedom provided a parsimonious balance between flexibility and stability, as indicated by improved predictive accuracy metrics and information criteria. This choice is also consistent with standard DLNM applications in environmental epidemiology (17, 18). Knot placement in both the exposure and lag dimensions followed the default quantile-based specification of the natural cubic spline function implemented in the dlnm package (i.e., internal knots placed at equally spaced quantiles of the observed distribution), without manual tuning.

We also fitted these models without the lagged and nonlinear specifications, that is, as generalized linear models with random effects for all specified distributions (GLM-Poisson_*rf*_, GLM-NegBin_*rf*_ and GLM-ZIP_*rf*_), in order to evaluate the improvement of incorporating the DLNM framework.

Model selection was based on information criteria (AIC, BIC) (53–57), and accuracy measures, including mean absolute error (MAE), and root mean square error (RMSE) (58, 59, 59–63). The RMSE defined as follows


$$\begin{array}{l} \text{RMSE}=\sqrt{\frac{1}{Tn}\underset{i,t}{\sum }{\left(Y_{i,t}-{\hat{Y}}_{i,t}\right)}^{2}}, \end{array}$$

where *t* = 1, ..., *T* denotes the number of weeks for *i* = 1, ..., *n* subregion in each climatic region; *Y*_*i, t*_ and ${\hat{Y}}_{i,t}$ are the observed and estimated hospital discharges, respectively, for subregion *i* at week *t*. The MAE is computed as follows:


$$\begin{array}{l} \text{MAE}=\frac{1}{Tn}\overset{m}{\underset{i,t}{\sum }}|Y_{it}-{\hat{Y}}_{it})|. \end{array}$$

We also incorporate a composite scoring methodology following the principles of rganisation for Economic Co-operation and Development (OECD) (64), where normalization is essential for ensuring comparability among heterogeneous indicators. Denote by *m*_*i, j*_ the value of the *j*-th performance metric (MAE, RMSE, AIC, BIC) for model *i*. Two normalization schemes were adopted: (i) the *min–max* normalization, defined as


$$z_{i,j}^{\left(1\right)}=1-\frac{m_{i,j}-\min\left(m_{j}\right)}{\max\left(m_{j}\right)-\min\left(m_{j}\right)},$$

which rescales all metrics into the interval [0, 1] with inverse orientation (smaller values implying better performance); and (ii) the *z-score* standardization,


$$z_{i,j}^{\left(2\right)}=-\frac{m_{i,j}-{\bar{m}}_{j}}{s_{j}},$$

where ${\bar{m}}_{j}$ and *s*_*j*_ are the mean and standard deviation of metric *j*, respectively. For each model *i*, the overall composite score was obtained as the arithmetic mean across all normalized indicators:


$$S_{i}^{\left(k\right)}=\frac{1}{p}\overset{p}{\underset{j=1}{\sum }}z_{i,j}^{\left(k\right)},$$

where *k* = 1, 2, and *p* denotes the number of metrics considered. We refer to $S_{i}^{\left(1\right)}$ and $S_{i}^{\left(2\right)}$ as the *Min-max score* and the *Z-score*, respectively. This normalization-based aggregation enhances interpretability and reduces implicit weighting biases among indicators (65). Sensitivity analysis was performed to compare the two composite scores, revealing a strong linear association (*r*>0.9), which confirms the robustness of the ranking and the internal consistency of the derived indicators. In addition, raw residuals, Pearson residuals, and deviance residuals were inspected; among competing model specifications, those minimizing residual autocorrelation were prioritized to ensure the model captures underlying systematic temporal patterns (28, 66–68).

Finally, exposure-lag surfaces and relative risk curves with 95% confidence intervals were reported for interpretation (43, 47). In addition, cumulative incidence rate ratios (IRRs) were derived from the fitted DLNM by aggregating lag-specific contributions across the entire lag period and exponentiating the corresponding linear predictor, following the standard DLNM framework (17, 43). To account for potential nonlinear and asymmetric exposure–response relationships, percentile-based contrasts were defined by comparing the 95th and 5th percentiles of each exposure to the regional median (50th percentile), as commonly applied in environmental epidemiology studies using distributed lag models (69, 70). Confidence intervals were obtained from the model-based variance–covariance matrix.

All analyses were conducted using the open-source software R (version 4.4.3) (71). The DLNM with random effects was fitted using the dlnm package (version 2.4.10) (43), while the zero-inflated versions were implemented within the Generalized Additive Model for Location, Scale, and Shape (GAMLSS) framework using the gamlss package (version 5.5-0) (27, 28).

## 3 Results

Table 3 reports the maximum lag for each covariate in across regions, based on an exploratory correlation analysis using Pearson correlation, distance correlation, and mutual information to capture both linear and nonlinear relationships. A higher lag value indicates more persistent lagged associations; therefore, it is important to include variables up to those lags in the model. Similar patterns are observed across subregions within each region (see Supplementary Tables S1 and Supplementary Figures S2, S3 for results by subregion).

**Table 3:** Maximum lag (*L*) selected for each climatic exposure (temperature, precipitation, relative humidity, and AOD) in the DLNM across climatic regions, based on the correlation analysis (Pearson, distance correlation, and mutual information) between each exposure and the hospitalization.

Exposure	PC	PN	PS	RN	RA	RMS
Temperature	9	14	11	7	5	14
Precipitation	9	6	7	4	12	11
Relative humidity	9	8	7	4	7	13
AOD	11	13	13	11	11	11

Across all exposures, nonlinear dependence predominated at the most pronounced lags, where significant Pearson correlations were comparatively smaller in magnitude than distance correlation and mutual information values, further supporting the need for a nonlinear lag specification.

By using the specification of the relevant lag structure and degree of nonlinearity for each climatic exposure, we fit all described models. Table 4 compares the goodness of fit of all fitted models for each region. The composite scores indicate that the DLNM with random effects and a negative binomial distribution (DLNM-NegBin_*rf*_) achieved the highest normalized performance in most peripheral climatic regions (PC, PN, PS, and RN), reflecting a superior balance between model fit and parsimony.

**Table 4:** Goodness-of-fit metrics (predictive accuracy, information criteria, and composite scores) of different fitted models for hospital discharge in six peripheral regions.

Region	Model	MAE	RMSE	AIC	BIC	*Min–Max score*	*Z-score*
PC	GLM-Poisson_*rf*_	1.785	2.558	12,398.460	12,736.250	0.029	–1.676
	GLM-NegBin_*rf*_	1.780	2.608	11,732.010	12,075.840	0.222	–1.145
	DLNM-ZIP	1.469	2.210	10,992.310	11,529.160	0.898	0.444
	DLNM-ZINBI	1.476	2.237	10,825.780	11,368.670	0.931	0.542
	DLNM-Poisson_*rf*_	1.450	2.182	10,915.300	11,452.150	0.954	0.580
	DLNM-NegBin_*rf*_	1.455	2.202	10,771.750	11,314.630	0.985	0.670
	DLNM-ZIP_*rf*_	1.450	2.183	10,898.830	11,453.730	0.955	0.585
PN	GLM-Poisson_*rf*_	3.499	5.129	23,541.300	23,901.530	0.028	–1.693
	GLM-NegBin_*rf*_	3.513	5.157	22,520.000	22,886.540	0.416	–0.605
	DLNM-ZIP	3.267	4.721	22,843.380	23,405.830	0.706	0.030
	DLNM-ZINBI	3.286	4.774	22,204.150	22,772.920	0.919	0.640
	DLNM-Poisson_*rf*_	3.247	4.693	22,821.550	23,384.000	0.749	0.136
	DLNM-NegBin_*rf*_	3.270	4.748	22,204.880	22,773.660	0.948	0.709
	DLNM-ZIP_*rf*_	3.247	4.692	22,802.780	23,390.420	0.751	0.144
	DLNM-ZINBI_*rf*_	3.287	4.774	22,204.160	22,772.990	0.918	0.638
PS	GLM-Poisson_*rf*_	3.187	4.673	27,386.570	27,753.030	0.070	–1.666
	GLM-NegBin_*rf*_	3.210	4.726	26,487.680	26,860.680	0.277	–0.852
	DLNM-ZIP	3.047	4.528	26,583.890	27,166.290	0.556	–0.206
	DLNM-ZINBI	3.050	4.500	26,399.690	26,988.630	0.633	0.050
	DLNM-Poisson_*rf*_	2.974	4.436	26,245.750	26,828.150	0.818	0.571
	DLNM-NegBin_*rf*_	2.981	4.459	25,654.000	26,242.950	0.973	1.154
	DLNM-ZIP_*rf*_	2.974	4.437	26,218.870	26,833.930	0.820	0.579
	DLNM-ZINBI_*rf*_	3.015	4.462	26,253.590	26,874.640	0.743	0.371
RA	GLM-Poisson_*rf*_	4.292	5.770	33,765.130	34,131.530	0.135	–1.705
	GLM-NegBin_*rf*_	4.467	6.106	30,928.610	31,301.550	0.302	–1.129
	DLNM-ZIP	3.770	5.173	30,577.350	31,159.660	0.770	0.095
	DLNM-ZINBI	3.808	5.228	29,244.000	29,832.860	0.887	0.466
	DLNM-Poisson_*rf*_	3.663	5.070	30,130.690	30,713.000	0.876	0.391
	DLNM-NegBin_*rf*_	3.678	5.096	28,976.190	29,565.050	0.988	0.740
	DLNM-ZIP_*rf*_	3.664	5.071	30,083.320	30,698.320	0.879	0.400
	DLNM-ZINBI_*rf*_	3.678	5.096	28,950.060	29,571.590	0.989	0.743
RMS	GLM-Poisson_*rf*_	1.800	2.292	9,157.490	9,478.260	0.166	–1.067
	GLM-NegBin_*rf*_	1.801	2.294	9,095.490	9,421.890	0.354	–0.480
	DLNM-ZIP	1.737	2.227	9,073.770	9,574.620	0.527	–0.263
	DLNM-ZINBI	1.736	2.228	9,041.210	9,547.690	0.626	0.043
	DLNM-Poisson_*rf*_	1.721	2.204	9,042.990	9,543.840	0.743	0.318
	DLNM-NegBin_*rf*_	1.722	2.206	9,015.580	9,522.060	0.816	0.549
	DLNM-ZIP_*rf*_	1.722	2.205	9,033.980	9,545.990	0.749	0.339
	DLNM-ZINBI_*rf*_	1.722	2.206	9,008.770	9,526.370	0.820	0.562
RN	GLM-Poisson_*rf*_	2.598	3.389	26,935.300	27,301.700	0.023	–1.673
	GLM-NegBin_*rf*_	2.606	3.394	26,231.330	26,604.280	0.361	–0.669
	DLNM-ZIP	2.511	3.266	26,466.560	27,048.570	0.550	–0.320
	DLNM-ZINBI	2.515	3.272	26,000.700	26,589.550	0.771	0.338
	DLNM-Poisson_*rf*_	2.476	3.224	26,282.280	26,864.590	0.774	0.262
	DLNM-NegBin_*rf*_	2.478	3.227	25,846.510	26,435.370	0.988	0.895
	DLNM-ZIP_*rf*_	2.477	3.224	26,254.970	26,869.820	0.777	0.272
	DLNM-ZINBI_*rf*_	2.478	3.227	25,830.140	26,451.240	0.987	0.894

In contrast, the zero-inflated variant (DLNM-ZINBI_*rf*_) exhibited slightly higher scores in regions RA and RMS, where overdispersion and structural zeros are more pronounced. These results suggest that negative binomial mixed specifications provide the most robust and stable modeling framework overall, while zero-inflated extensions offer localized improvements under data sparsity or excess-zero conditions.

The inclusion of nonlinear lagged effects of climatic covariates improved model fit. Moreover, subregional random effects adequately captured unexplained spatial heterogeneity, reducing residual variance and further enhancing overall model performance. This advantage was consistent compared to alternatives without random effects, which exhibited overdispersion in the response variable. The performance of the best-fitting model for each region was also evaluated through visual inspection of the Pearson residuals (for more details, see Supplementary Figures S10–S15). Although the residuals of the fitted models in some subregions still exhibited autocorrelation, we consider them appropriate for the subsequent analysis.

The main advantage of the DLNM framework lies in its ability to represent the lagged and nonlinear effects of each covariate by estimating the exposure-response-lag relationship. These effects were obtained from the best-fitting model for each region by exponentiating the covariate effects derived from the product of the cross-basis functions. These values were compared with a reference value (their average) to quantify the relative risk (RR) associated with increases in hospital discharges.

**Figure 2:**
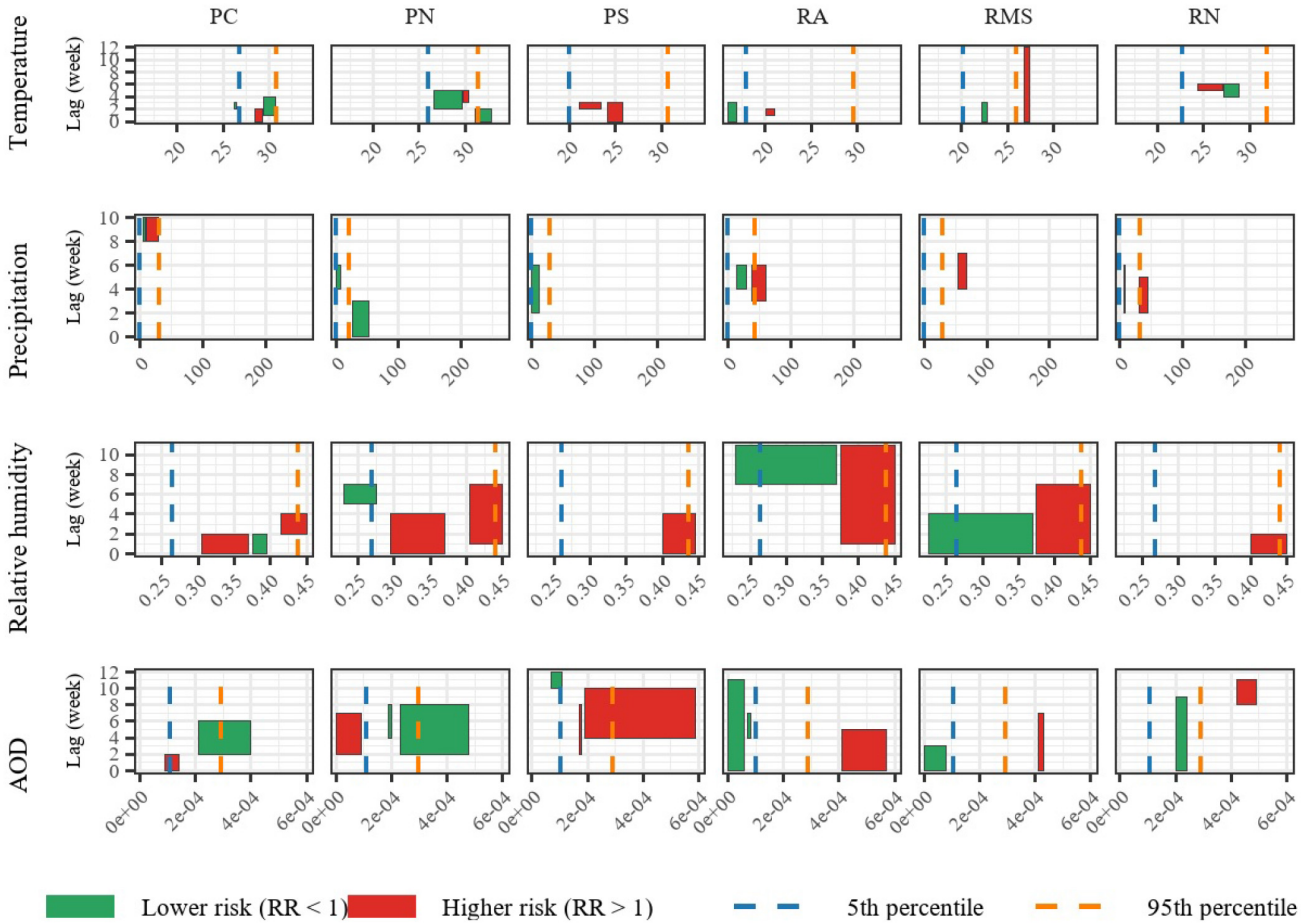
Statistically significant lag periods and exposure ranges at a 5% significance level for each climatic exposure (temperature, precipitation, relative humidity, and AOD) across regions obtained by the best-fitting model. Colored rectangles denote significant exposure–lag combinations: green indicates lower risk (< 1), and red indicates higher risk (>1). Blue and orange dashed lines represent the 5th and 95th percentiles, respectively, of each climatic exposure for the subregions within each region.

For each climatic region, we extract the relevant lags and exposure ranges for each covariate at 5% significance level, and the summaries are visualized in Figure 2 (For precise ranges for each covariate across regions, see Supplementary Tables S3, S4, and the three-dimensional surfaces, contour maps, and lag-response curves of the selected model across the six regions can be found in Supplementary Figures S4–S9). Colored rectangles denote exposure-lag combinations with relative risks (RR) significantly different from 1 at the 5% level. Green indicates decreased risk (*RR* < 1) and red increased risk (*RR*>1). The horizontal and vertical extents represent exposure ranges and lag (weeks), respectively. The 5th (blue dashed line) and 95th (orange dashed line) percentiles of each climatic exposure for each region are included to facilitate meaningful interpretation (For more details on the descriptive statistics of the climatic exposures, see Supplementary Table S2).

Cumulative effects were derived from the DLNM–GLMM by integrating lag-specific contributions across the entire lag period, yielding cumulative incidence rate ratios (IRRs) as measures of the total relative risk associated with a given exposure level. Because climatic exposure–response relationships are typically non-linear and potentially asymmetric, percentile-based contrasts were used instead of linear increments. Specifically, cumulative IRRs were computed comparing the 95th percentile to the median (high exposure contrast) and the 5th percentile to the median (low exposure contrast). This bidirectional percentile approach enables a balanced assessment of risk associated with both elevated and reduced climatic conditions and is consistent with recent environmental epidemiology studies summarizing non-linear climate-related health impacts using distributed lag models (69, 70). The resulting regional cumulative IRRs, along with their corresponding confidence intervals and statistical significance, are presented in Table 5.

**Table 5:** Regional cumulative incidence rate ratios (IRR) comparing extreme percentiles against the median.

Region	Variable	Contrast	IRR	CI_*low*_	CI_*high*_	*p*
RN	T	p95 vs. p50	1.022	0.902	1.158	0.7338
		p5 vs. p50	1.154	1.029	1.294	0.0141
	P	p95 vs. p50	0.984	0.883	1.097	0.7712
		p5 vs. p50	1.035	0.932	1.149	0.5236
	RH	p95 vs. p50	1.153	1.092	1.218	0.0000
		p5 vs. p50	1.049	0.962	1.143	0.2771
	AOD	p95 vs. p50	0.768	0.726	0.813	0.0000
		p5 vs. p50	1.157	1.099	1.219	0.0000
RA	T	p95 vs. p50	1.172	0.775	1.773	0.4524
		p5 vs. p50	1.467	0.901	2.389	0.1237
	P	p95 vs. p50	1.155	0.984	1.357	0.0788
		p5 vs. p50	1.097	1.049	1.147	0.0001
	RH	p95 vs. p50	1.556	1.411	1.717	0.0000
		p5 vs. p50	0.832	0.754	0.918	0.0003
	AOD	p95 vs. p50	0.802	0.765	0.840	0.0000
		p5 vs. p50	0.922	0.881	0.965	0.0005
PC	T	p95 vs. p50	0.855	0.749	0.977	0.0215
		p5 vs. p50	0.714	0.534	0.955	0.0232
	P	p95 vs. p50	1.009	0.736	1.383	0.9575
		p5 vs. p50	0.968	0.628	1.492	0.8820
	RH	p95 vs. p50	1.357	1.193	1.543	0.0000
		p5 vs. p50	1.075	0.889	1.301	0.4557
	AOD	p95 vs. p50	0.847	0.718	1.000	0.0495
		p5 vs. p50	1.031	0.840	1.266	0.7685
PS	T	p95 vs. p50	0.694	0.605	0.796	0.0000
		p5 vs. p50	0.574	0.447	0.737	0.0000
	P	p95 vs. p50	0.970	0.878	1.072	0.5551
		p5 vs. p50	0.750	0.654	0.860	0.0000
	RH	p95 vs. p50	1.174	1.095	1.259	0.0000
		p5 vs. p50	1.056	0.931	1.198	0.3958
	AOD	p95 vs. p50	0.908	0.845	0.976	0.0089
		p5 vs. p50	0.971	0.896	1.053	0.4774
PN	T	p95 vs. p50	0.993	0.963	1.023	0.6294
		p5 vs. p50	0.824	0.726	0.936	0.0029
	P	p95 vs. p50	1.018	0.917	1.130	0.7416
		p5 vs. p50	0.825	0.713	0.954	0.0094
	RH	p95 vs. p50	1.739	1.579	1.916	0.0000
		p5 vs. p50	0.906	0.785	1.047	0.1803
	AOD	p95 vs. p50	0.824	0.774	0.876	0.0000
		p5 vs. p50	1.127	1.047	1.214	0.0016
RMS	T	p95 vs. p50	1.199	1.015	1.416	0.0327
		p5 vs. p50	0.688	0.534	0.888	0.0040
	P	p95 vs. p50	1.105	0.911	1.340	0.3118
		p5 vs. p50	0.931	0.808	1.074	0.3256
	RH	p95 vs. p50	2.048	1.620	2.590	0.0000
		p5 vs. p50	0.574	0.354	0.932	0.0248
	AOD	p95 vs. p50	0.768	0.663	0.890	0.0004
		p5 vs. p50	1.039	0.879	1.228	0.6525

IRR denotes cumulative incidence rate ratio. p95 and p5 correspond to the 95th and 5th percentiles of each exposure relative to the regional median (p50). CI low and CI high correspond to the lower and upper bounds of the 95% confidence interval.

Across climatic regions, we summarize these findings as follows:

- PC and PN share similar patterns in almost all variables since both belong to the North and Central Pacific, with similar climate dynamics.
- PS presents a more pronounced relative risk for medium or high pollution levels, which persist for 5–10 weeks, a considerable difference from the other Pacific regions.
- The RA, RMS, and RN regions show heterogeneous responses in climate and pollution variables. They exhibit higher precipitation levels than the other peripheral regions, associated with an increased risk of hospital discharges for respiratory illnesses. This is consistent with the climatic characteristics of these three regions, which are characterized by more intense rainfall and cooler microclimates.

Regarding to the temperature, the intervals are small and dispersed; temperature effects do not appear to dominate the weekly variability of discharges. However, the RMS region, which generally has lower temperatures than the other regions, shows a high relative risk near 27 °C for a prolonged period of 0 to 12 weeks. This suggests that averaged weekly temperature alone does not accurately capture the relevant physiological exposure (minimum temperature or temperature range would probably be more informative).

In terms of precipitation, in RA, RMS, and RN, high values (above the 95th percentile) are associated with higher risk, reflecting the impact of intense rainfall events, which are typically linked to increased ambient humidity. At low precipitation levels, the protective effect may be due to greater atmospheric stability and fewer fluctuations in relative humidity. PC region presents a high risk at medium values (10-30 mm), which are medium to high precipitation in this region and a drier climate is common. This could reflect transitional episodes (beginning or end of the rainy season), where there is high variability and particles resuspended by the first rain (72).

On the other hand, high RH values (>0.43) coincide with higher risks, reflecting saturated environments in which respiratory illnesses tend to increase. In PC and PN, average relative humidity values (0.36 − 0.40) are also associated with elevated risk, possibly due to the influence of Pacific maritime conditions, exposure to which has been shown to have detrimental effects on human health (73, 74). Low RH values (< 0.27) are consistently protective, as drier conditions reduce the survival of biological agents and hygroscopic particles (75).

In terms of statistically significant cumulative effects, exposure to low temperatures (p5 vs. p50) was associated with a 15.4% increased risk in RN (IRR = 1.154, *p* = 0.0141), while significant protective effects were observed in PC, PS, PN, and RMS. Exposure to high temperatures (p95 vs. p50) showed significant protective effects in PC and PS, but an increased risk in RMS. Relative humidity exhibited the most consistent and strongest positive associations across regions, particularly in RA, PC, PS, PN, and RMS, where high exposure contrasts produced IRRs substantially greater than 1. Aerosol optical depth (AOD) showed significant inverse associations at high percentiles in most regions, whereas low AOD levels were associated with increased risk in RN and PN. In contrast, precipitation effects were generally weaker and less consistent, although significant associations were detected in selected regions for low exposure contrasts.

Finally, high AOD values imply greater risk of respiratory hospitalization in most regions, reflecting the fine aerosol load and its relationship with respiratory illnesses. In PC and PN, however, the opposite pattern is observed. This could be explained by two factors related to thermal inversion and atmospheric stability during the dry season: (1) low AOD measured by satellite despite high local surface concentrations due to limited vertical mixing, and (2) the presence of local non-optically active pollutants, such as ozone or gases, that do not increase AOD but do increase risk (76). This suggests that in the Pacific, satellite-detected aerosols may underestimate surface concentrations during dry and stable periods.

## 4 Discussion

We proposed a methodological framework based on the DLNM that integrates subregional random effects, zero-inflated distributions, and nonlinear and delayed effects of climatic and pollution variables to model weekly hospital discharges due to respiratory causes in Costa Rica. The results confirm that DLNM with count distribution with subregional random effects capture nonlinear and lagged relationships between climate, pollution, and respiratory morbidity/mortality in high-variability contexts. In particular, we identified critical risk-increase windows associated with environmental variables, with heterogeneity within regions and by exposure level.

The regional heterogeneity observed in the exposure-response relationships is likely driven by climatic, environmental, and demographic differences across Costa Rican subregions. Previous studies have shown that the health impacts of temperature and precipitation depend strongly on local baseline climate conditions, population adaptation, and infrastructure characteristics (7, 69). For instance, warmer regions may exhibit attenuated heat effects due to long-term acclimatization, whereas cooler or highland regions may show stronger relative risks for equivalent temperature deviations. Similar regional variability has been documented in respiratory morbidity studies using distributed lag models (9, 12, 13).

In Costa Rica, climatic gradients between coastal and inland regions are well established (16), and previous national studies have reported spatially heterogeneous associations between climate variables and health outcomes (15, 77). Differences in humidity patterns, precipitation regimes, and aerosol composition (72, 78) may partly explain why certain regions exhibited stronger cumulative IRRs at extreme percentiles. These findings are consistent with the broader environmental epidemiology literature, which emphasizes that exposure-lag-response associations are context-specific and influenced by regional climatic baselines and adaptive capacity (19, 79).

The diversity of results reported in the literature may be attributed to differences in climatic conditions, data quality, study designs, and analytical methods. Nevertheless, the findings of our study are consistent with those of some previous works. An additional consideration is that in peripheral regions of Costa Rica, as in several tropical countries, cold weather is generally perceived as comfortable compared to temperate regions. Conversely, during periods of extreme heat, people tend to spend more time indoors to maintain comfort, often relying on artificial cooling systems.

These patterns are consistent with evidence from tropical/subtropical climates reporting nonlinear and delayed environmental effects on respiratory outcomes, strong spatial heterogeneity of associations, and the usefulness of hierarchical models with zero-inflated distributions when structural zeros and temporal dependence are present. The superiority of models with random effects over non-hierarchical alternatives has also been documented for improving estimate stability and the identification of regional thresholds.

Although the results cannot be interpreted as causal, the nonlinear lagged associations detected in this study are consistent with the existing literature. The exposure-lag thresholds enable the translation of model findings into surveillance and operational alerts; however, their direct applicability to policy decisions requires careful consideration. We recommend (1) defining activation thresholds based on extreme percentiles (e.g., P90/P95) of climatic exposures by subregion; (2) prioritizing peripheral regions with limited access to health services; (3) using hospital discharges as a sensitive indicator for detecting weekly peaks and standardizing monitoring; and (4) integrating these models into epidemiological surveillance systems to trigger preventive actions and guide resource allocation.

Our findings are consistent with previous studies conducted in Latin America examining climate–health associations. In Colombia, Rúa et al. (80) reported significant lagged associations between local climatic variability and dengue transmission using time-series models. Similarly, Vásquez et al. (81) identified nonlinear relationships between temperature and precipitation with dengue incidence in Costa Rica. Although these investigations focused on vector-borne diseases rather than respiratory outcomes, they consistently demonstrated that climatic exposures exert region-dependent and temporally lagged effects on health indicators, reinforcing the relevance of modeling delayed and nonlinear exposure–response relationships in tropical contexts.

Beyond cumulative incidence rate ratios, an important contribution of the present study lies in the identification of statistically significant exposure thresholds associated with relative risks above and below unity. By jointly examining exposure level, lag structure, and changes in hospital discharges, we were able to characterize regions of increased and decreased risk within the exposure–lag surface. This approach allows for a more nuanced interpretation than single-point estimates, as it captures the interaction between intensity of exposure and temporal displacement effects, consistent with distributed lag non-linear modeling frameworks (19, 79).

More broadly, studies conducted in tropical and subtropical settings have emphasized the importance of accounting for local climatic regimes when evaluating climate-sensitive health outcomes (10, 11). The presence of region-specific thresholds and heterogeneous lag structures in our results supports the notion that climatic baselines and adaptive conditions modulate health responses. In heterogeneous environments such as Costa Rica, percentile-based contrasts provide a scale-invariant framework for comparing effects across subregions while preserving the interpretation of relative risk in epidemiological terms.

The cumulative results highlight substantial regional heterogeneity in climate-health associations. Relative humidity emerged as the most consistent risk factor, suggesting that sustained high humidity conditions may play a critical role in respiratory morbidity in tropical peripheral regions. Temperature effects showed asymmetry, with cold-related impacts predominating in some regions (e.g., RN, PN), while heat-related increases were evident in RMS. The inverse association observed for high AOD percentiles in several regions may reflect complex exposure dynamics or residual confounding related to atmospheric conditions. Overall, these findings reinforce the importance of modeling non-linear and lagged effects, as both elevated and reduced climatic conditions contribute differently to respiratory risk across regions.

Among the limitations encountered in this study: (1) the series built from administrative records and satellite sources may contain measurement error, omissions, or inconsistencies; although data cleaning and imputation procedures were applied, potential biases remain. (2) DLNM interpretation may be affected by multicollinearity and by parametrization decisions for the basis functions; these were guided by correlation criteria, information metrics, and predictive performance, without guaranteeing global optimality. (3) Extending the model to include random slopes to account for subregional climatic effects increases computational cost and the risk of non-convergence. (4) Although the DLNM design reduces residual autocorrelation, it does not eliminate it completely given the complexity of dependencies; thus, inferences should focus primarily on exposure-lag associations.

Taking these limitations into account, future work may include: (1) exploring more flexible spatio-temporal models for low-count hospitalizations; (2) integrating additional pollutants and socio-environmental variables; and (3) using data with higher spatio-temporal resolution to enhance predictive capacity and improve early detection of extremes.

## Data Availability

The original contributions presented in the study are publicly available. This data can be found here: https://github.com/Emanuelle-Parra/DLNM-GLMM-health.
