# Supplementary material for: Zero-inflated and distributed lag nonlinear models with random effects for assessing environmental impacts on respiratory health in peripheral regions of Costa Rica
Source: Front Public Health. 2026 Apr 8;14:1753511. doi: 10.3389/fpubh.2026.1753511 (PMC13099877; doi:10.3389/fpubh.2026.1753511)
Supplement: Supplementary file 1 [file Supplementary_file_1.pdf]

Supplementary Material

Zero-Inflated and Distributed Lag Nonlinear Models with Random Effects for Assessing Environmental Impacts on Respiratory Health in Peripheral Regions of Costa Rica

This document presents the supplementary materials that support the findings of the paper. The following acronyms are used throughout the document to denote the climatic regions:

- North Pacific Region (PN),
- Central Pacific Region (PC),
- South Pacific Region (PS),
- Southern Mountain Region (RMS),
- Central Valley Region (VC),
- Northern Region (RN), and
- Atlantic Region (RA).

The subsequent number denotes the climatic subregion within each climatic region. For example, PN1 indicates the first subregion of PN, as shown in Figure 1 of the main paper.

The climatic exposures are: Temperature (T), Precipitation (P), Relative humidity (RH), and Aerosol optical depth (AOD).

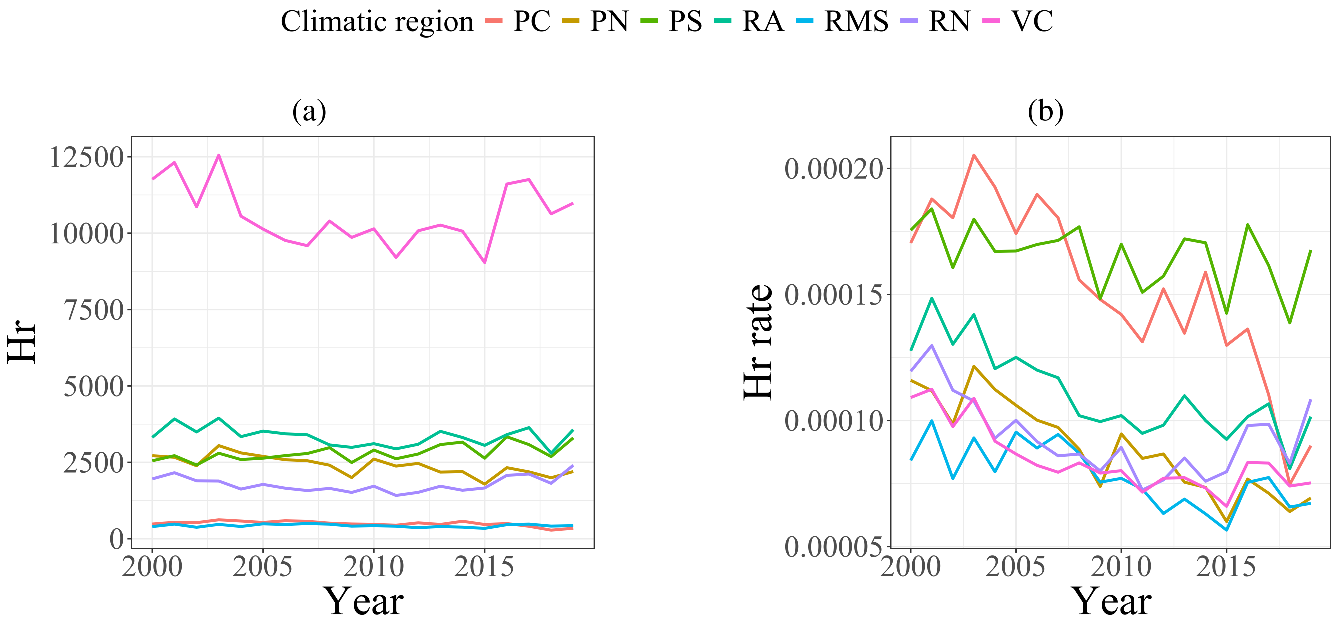

Figure S1. Annual total number of hospital discharges for respiratory diseases (Hr) (panel (a)) and the corresponding annual discharge rates (expressed per 100,000 population) (panel (b)) across the seven climatic regions of Costa Rica—Pacific Central (PC), Pacific North (PN), Pacific South (PS), Atlantic Region (RA), Metropolitan Region (RMS), Northern Region (RN), and Central Valley (VC)—from 2000 to 2019.

Table S1. Maximum lag for each climatic exposure—temperature, precipitation, relative humidity, and aerosol optical depth (AOD)—in the DLNM across climatic subregions, based on the correlation analysis between each exposure and hospital discharges.

Region	Subregion	Temperature	Precipitation	Relative humidity	AOD
PC	1	12	10	4	14
	2	9	7	9	14
	3	7	10	11	11
PN	1	5	6	7	9
	2	14	5	7	13
	3	6	5	9	13
	4	8	8	3	14
PS	1	3	6	6	12
	2	3	10	9	12
	3	12	8	10	11
	4	7	9	7	13
	5	13	7	8	10
RA	1	3	6	11	12
	2	5	12	9	13
	3	7	10	4	10
	4	3	4	6	13
	5	3	11	11	14
RMS	1	12	7	7	12
	2	12	11	13	12
RN	1	6	8	3	11
	2	7	10	3	9
	3	6	7	4	14
	4	11	7	7	11
	5	7	5	3	13
VC	1	12	3	4	13
	2	3	6	5	12
	3	9	5	4	10

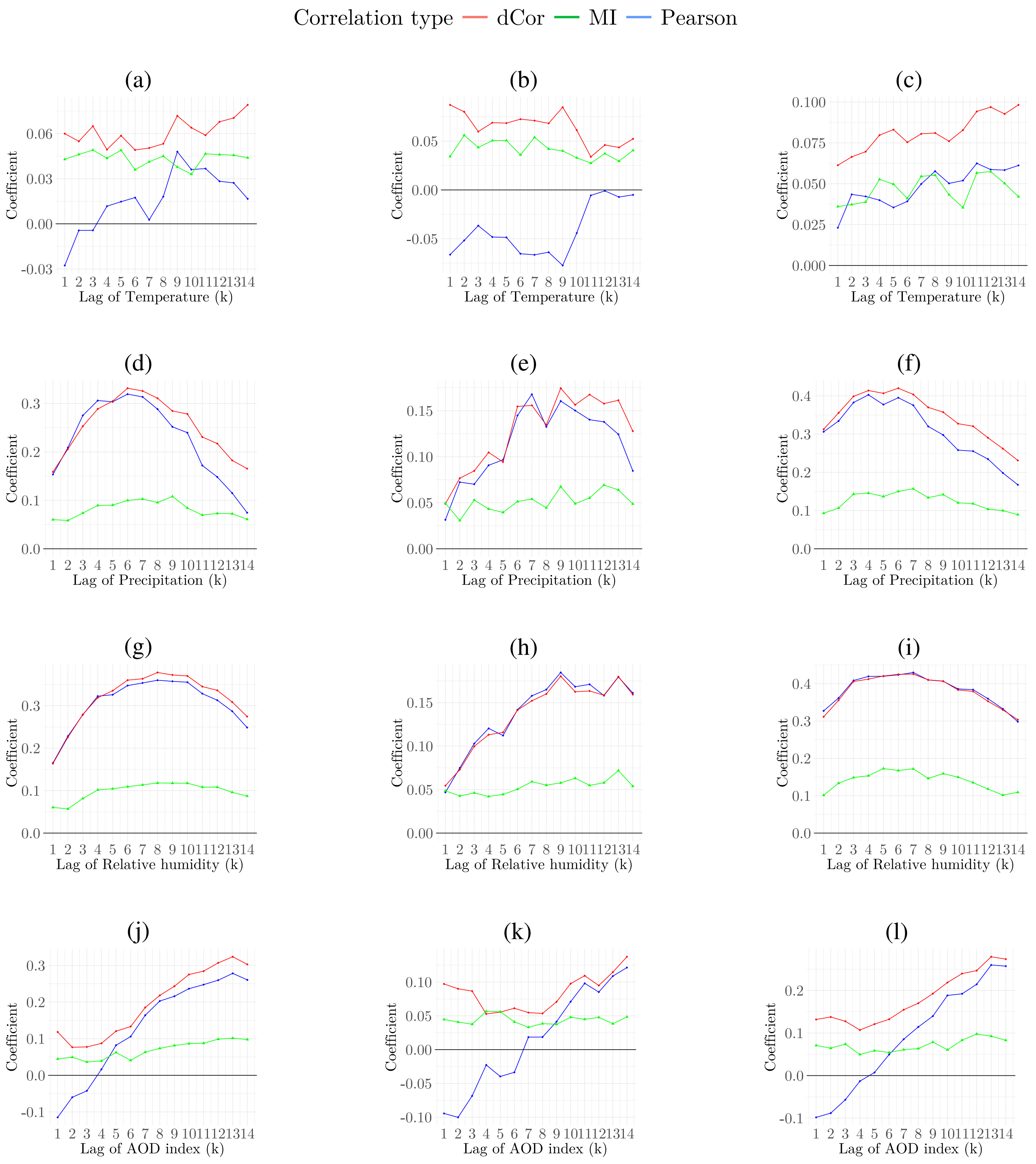

Figure S2. Pairwise correlations (Pearson's correlation coefficient, distance correlation, and mutual information) between weekly hospital discharges due to respiratory diseases and lagged climatic predictors in the Pacific subregions PN1, PC1, and PS1. Panels (a–c) correspond to temperature (T); (d–f) to precipitation (P); (g–i) to relative humidity (RH); and (j–l) to aerosol optical depth (AOD). The horizontal axis represents lag in weeks ($k = 1$–14).

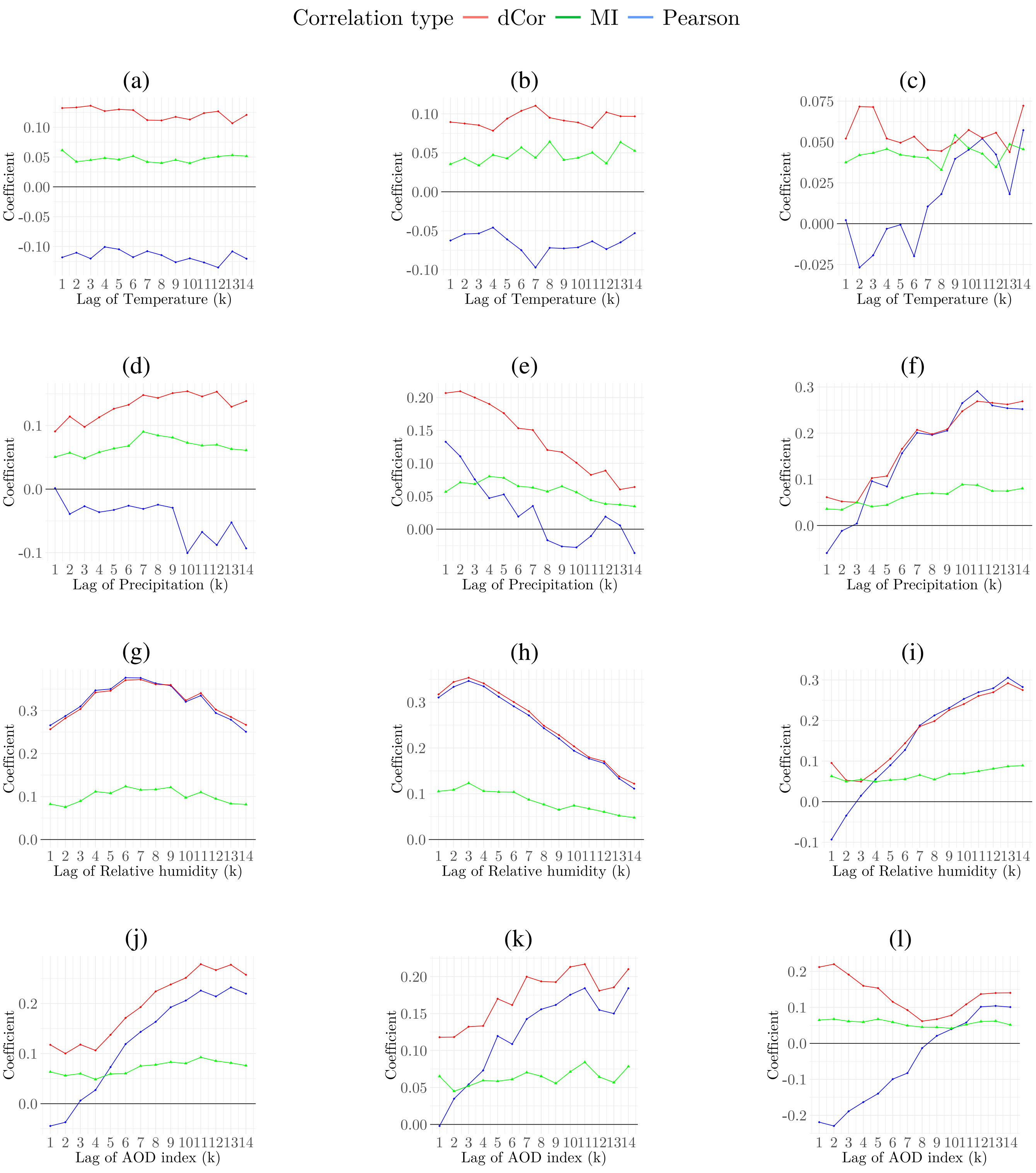

Figure S3. Pairwise correlations (Pearson's correlation coefficient, distance correlation, and mutual information) between weekly hospital discharges due to respiratory diseases and lagged climatic predictors in the Atlantic (RA1), Northern (RN1), and Southern Mountain (RMS1) subregions of Costa Rica. Panels (a–c) correspond to temperature (T); (d–f) to precipitation (P); (g–i) to relative humidity (RH); and (j–l) to aerosol optical depth (AOD). The horizontal axis represents lag in weeks ($k = 1$–14).

Table S2. Descriptive statistics for climatic exposure variables—temperature (T), precipitation (P), relative humidity (RH), and aerosol optical depth (AOD)— at the subregional levels across climatic regions.

Region	Variable	Mean	Minimum	Quartile 1	Median	Quartile 3	Maximum
PC	T	28.910	24.930	27.950	29.010	29.890	32.330
	P	12.284	0.000	3.511	11.007	17.805	71.292
	RH	0.370	0.222	0.316	0.396	0.422	0.451
	AOD	0.000192	0.000000	0.000147	0.000189	0.000229	0.000570
PN	T	28.940	23.350	27.910	29.150	30.040	32.860
	P	7.744	0.000	0.939	6.860	12.127	52.039
	RH	0.375	0.225	0.321	0.401	0.425	0.453
	AOD	0.000195	0.000000	0.000151	0.000191	0.000231	0.000509
PS	T	26.090	18.160	22.900	27.590	28.870	32.670
	P	13.066	0.000	6.002	12.377	17.897	96.102
	RH	0.368	0.219	0.311	0.393	0.420	0.450
	AOD	0.000189	0.000000	0.000142	0.000186	0.000226	0.000601
RA	T	25.010	15.950	20.860	27.270	28.490	31.080
	P	15.927	0.000	6.861	12.303	19.564	268.630
	RH	0.371	0.222	0.317	0.397	0.422	0.451
	AOD	0.000186	0.000000	0.000139	0.000183	0.000223	0.000607
RMS	T	23.190	18.690	21.620	23.000	24.960	27.410
	P	11.975	0.000	3.516	10.580	17.720	79.164
	RH	0.371	0.223	0.317	0.397	0.422	0.451
	AOD	0.000191	0.000000	0.000145	0.000188	0.000228	0.000568
RN	T	27.910	19.610	26.370	28.490	29.880	33.950
	P	12.880	0.000	4.616	11.023	17.075	182.235
	RH	0.375	0.225	0.322	0.401	0.425	0.452
	AOD	0.000191	0.000000	0.000146	0.000188	0.000227	0.000538

Table S3. Significant (5%) exposure–lag ranges and the corresponding types of significant relative risk (RR)—Low (RR < 1) and High (RR > 1)—for each climatic predictor (temperature (T), precipitation (P), relative humidity (RH), and aerosol optical depth (AOD)) from the best-fitted model across climatic subregions (PC, PN, and PS).

Region	Variable	Exposure range		Lag range		RR type
		Minimum	Maximum	Minimum	Maximum	
PC	Temperature	26.2	26.5	2	3	Low
		28.5	29.3	0	2	High
		29.4	30.7	1	4	Low
	Precipitation	6	9	8	10	Low
		10	29	8	10	High
	Relative humidity	0.305	0.370	0	2	High
		0.375	0.395	0	2	Low
		0.415	0.450	2	4	High
	AOD	0.00009	0.00014	0	2	High
		0.00021	0.00040	2	6	Low
PN	Temperature	26.6	29.7	2	5	Low
		29.8	30.4	3	5	High
		31.1	32.8	0	2	Low
	Precipitation	0	7	4	6	Low
		27	52	0	3	Low
	Relative humidity	0.230	0.275	5	7	Low
		0.295	0.370	0	4	High
		0.405	0.450	1	7	High
	AOD	0.00000	0.00009	2	7	High
		0.00019	0.00020	4	8	Low
		0.00023	0.00048	2	8	Low
PS	Temperature	21.1	23.4	2	3	High
		24.2	25.8	0	3	High
	Precipitation	0	12	2	6	Low
	Relative humidity	0.401	0.445	0	4	High
	AOD	0.00007	0.00011	10	12	Low
		0.00017	0.00018	2	8	High
		0.00019	0.00059	4	10	High

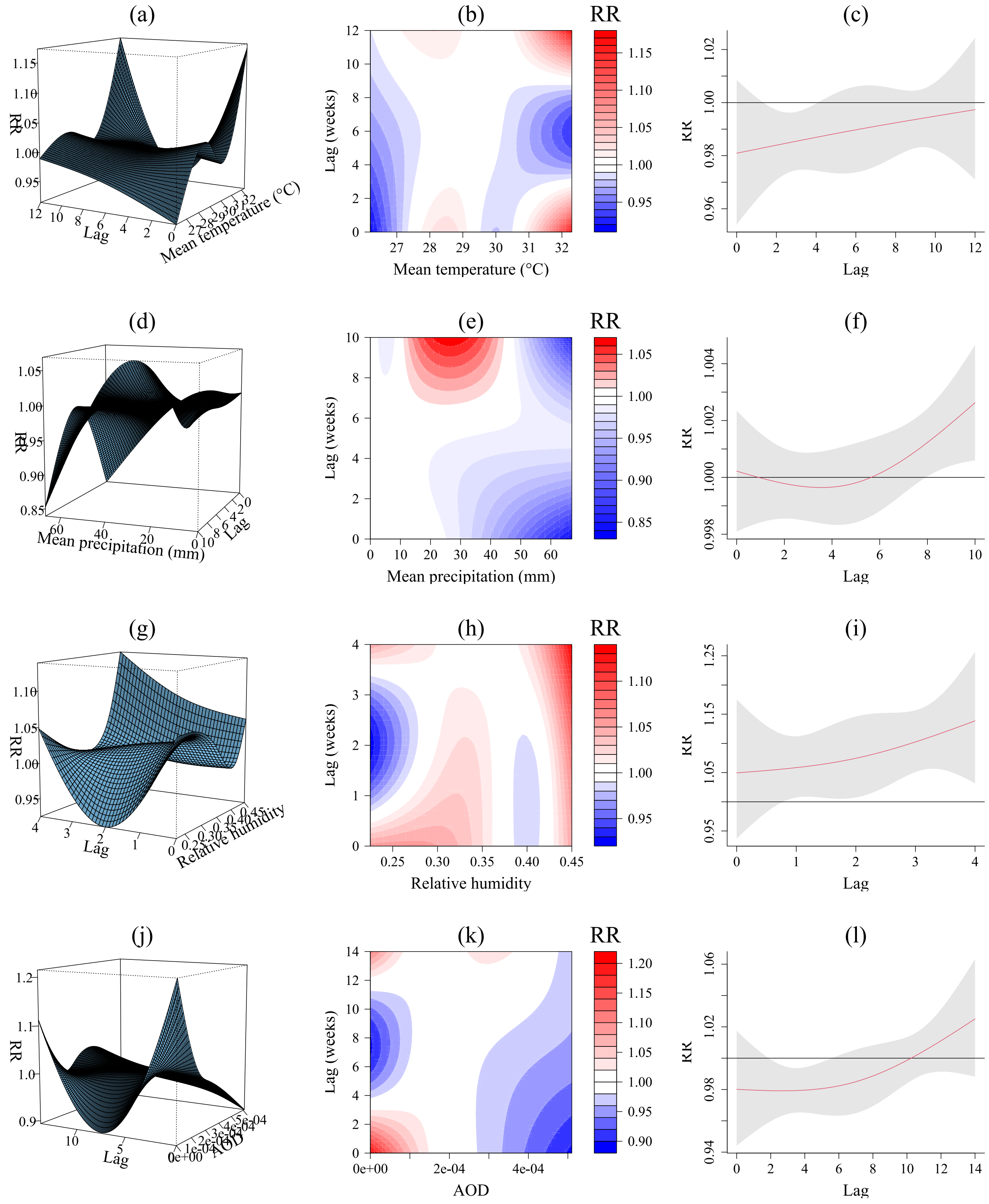

Figure S4. Exposure–lag–response associations between climatic predictors and the relative risk (RR) of weekly respiratory hospital discharges in PC1, estimated from the optimal distributed lag nonlinear mixed model (negative binomial with subregional random effects). Rows correspond to temperature (T, °C), precipitation (P, mm), relative humidity (RH), and aerosol optical depth (AOD). Panels (a,d,g,j) show three-dimensional surfaces; (b,e,h,k) contour plots; and (c,f,i,l) lag–response curves (lag in weeks). Colors indicate the magnitude of RR (red: RR >1; blue: RR <1). Shaded bands denote 95% confidence intervals. Predictions are centered at the subregional mean exposure.

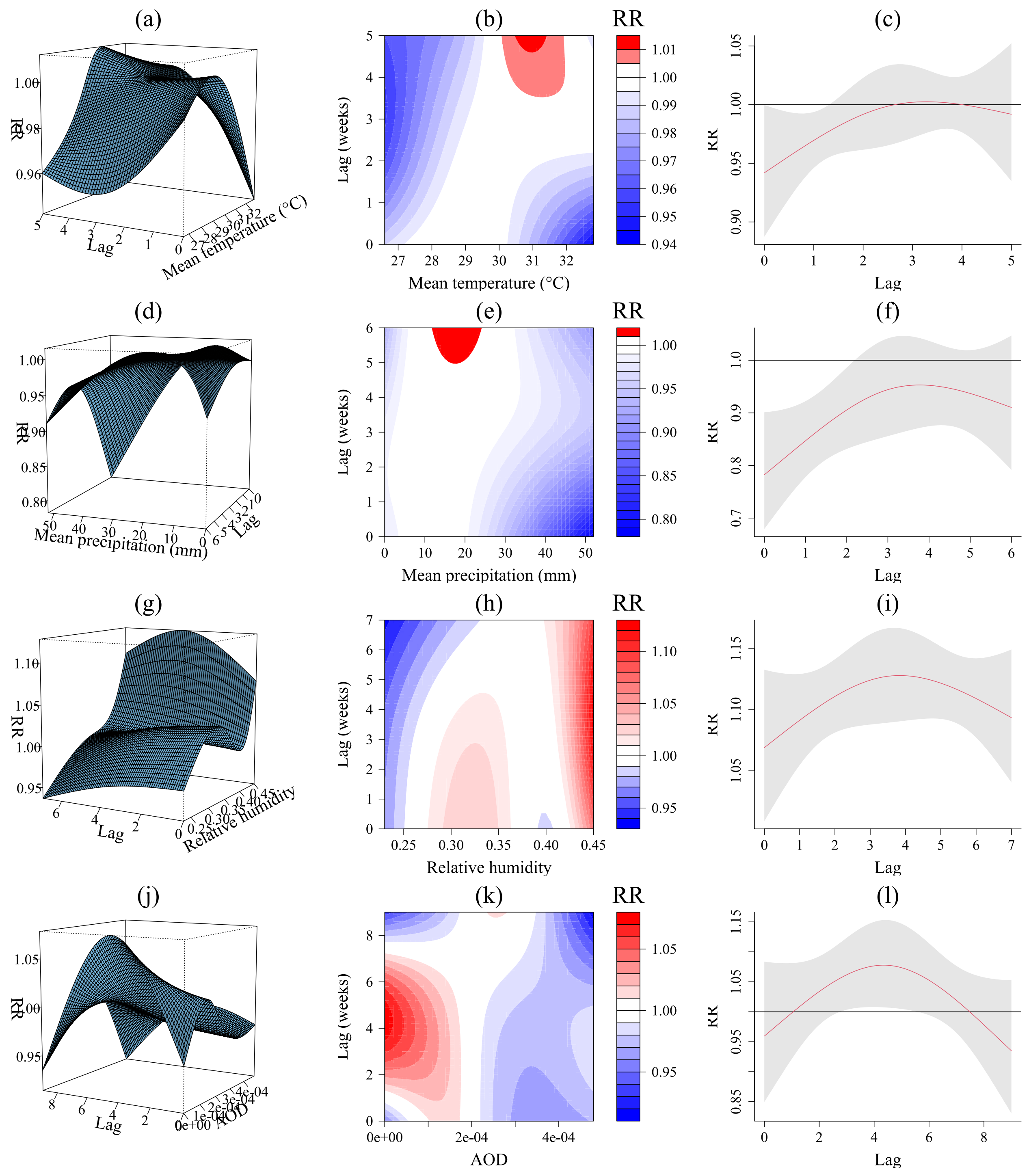

Figure S5. Exposure–lag–response associations between climatic and air-quality predictors and the relative risk (RR) of weekly respiratory hospital discharges in subregion PN1, estimated from the optimal distributed lag nonlinear mixed model (negative binomial with subregional random effects). Rows correspond to temperature (T), precipitation (P), relative humidity (RH), and aerosol optical depth (AOD). Panels (a,d,g,j) show three-dimensional surfaces; (b,e,h,k) contour plots; and (c,f,i,l) lag–response curves (lag in weeks). Colors represent the magnitude of RR (red: RR >1; blue: RR <1). Predictions are centered at the subregional mean exposure.

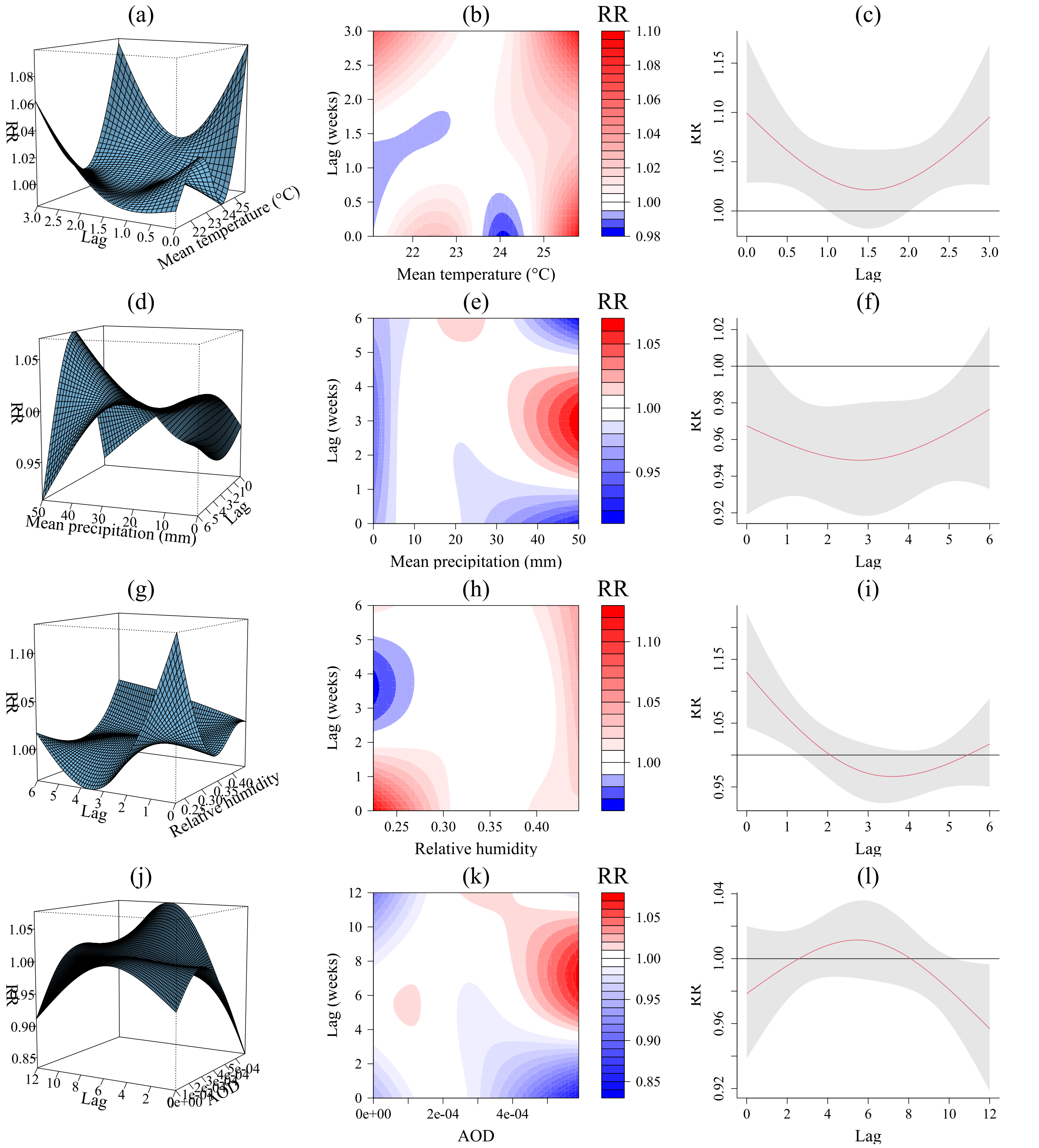

Figure S6. Exposure–lag–response associations between climatic and air-quality predictors and the relative risk (RR) of weekly respiratory hospital discharges in subregion PS1, estimated from the optimal distributed lag nonlinear mixed model (negative binomial with subregional random effects). Rows correspond to temperature (T), precipitation (P), relative humidity (RH), and aerosol optical depth (AOD). Panels (a,d,g,j) show three-dimensional surfaces; (b,e,h,k) contour plots; and (c,f,i,l) lag–response curves (lag in weeks) evaluated at representative exposure levels (observed maximum for T, observed minima for P and RH, and $AOD = 8 \times 10^{-5}$). Colors indicate the magnitude of RR (red: RR > 1; blue: RR < 1). Predictions are centered at the subregional mean exposure.

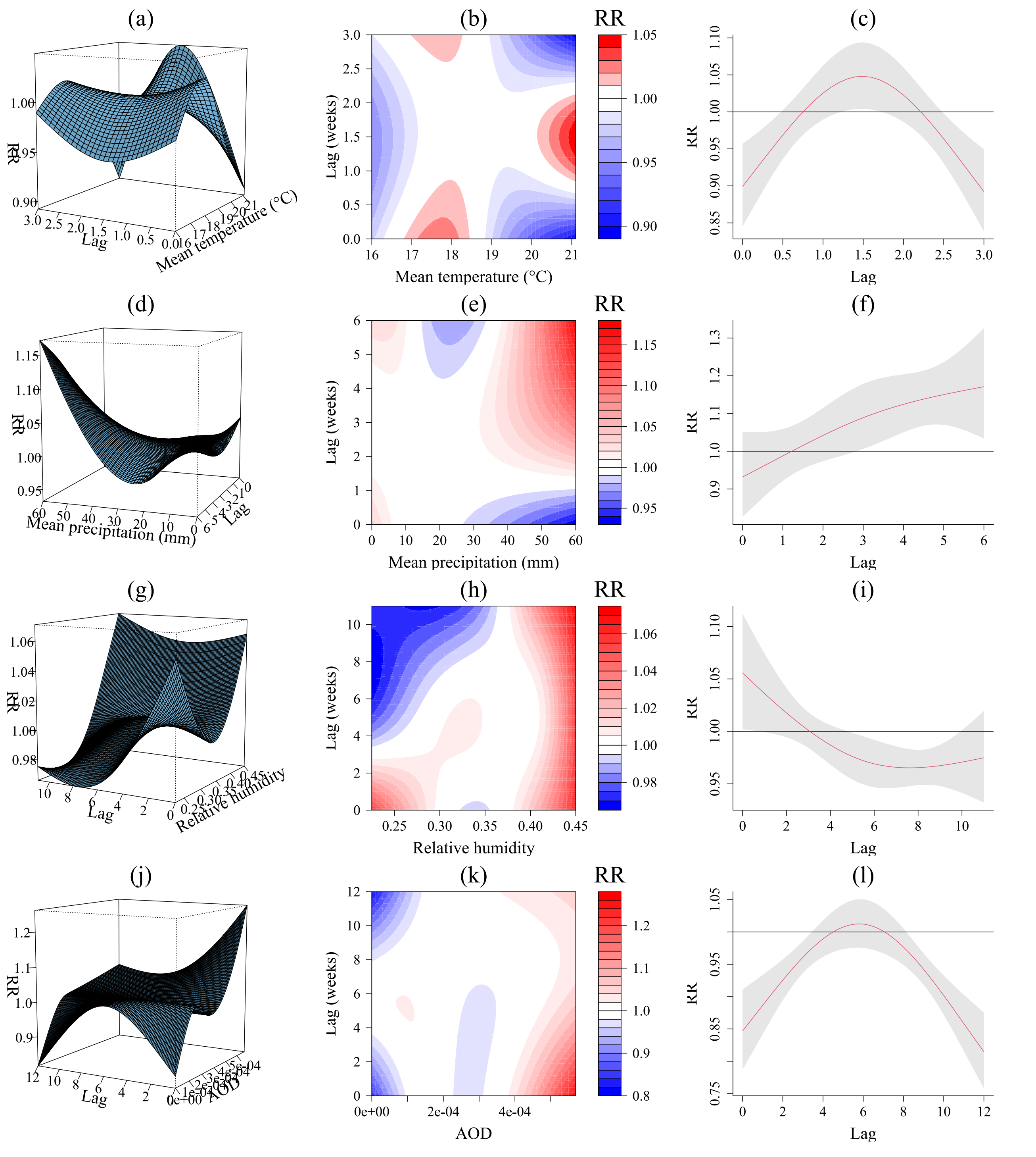

Figure S7. Exposure–lag–response associations between climatic and air-quality predictors and the relative risk (RR) of weekly respiratory hospital discharges in subregion RA1, estimated from the optimal distributed lag nonlinear mixed model (zero-inflated negative binomial with subregional random effects). Rows correspond to temperature (T), precipitation (P), relative humidity (RH), and aerosol optical depth (AOD). Panels (a,d,g,j) show three-dimensional surfaces; (b,e,h,k) contour plots; and (c,f,i,l) lag–response curves (lag in weeks) evaluated at representative exposure levels (observed maxima for T and P, and observed minima for RH and AOD). Colors indicate the magnitude of RR (red: RR > 1; blue: RR < 1). Predictions are centered at the subregional mean exposure.

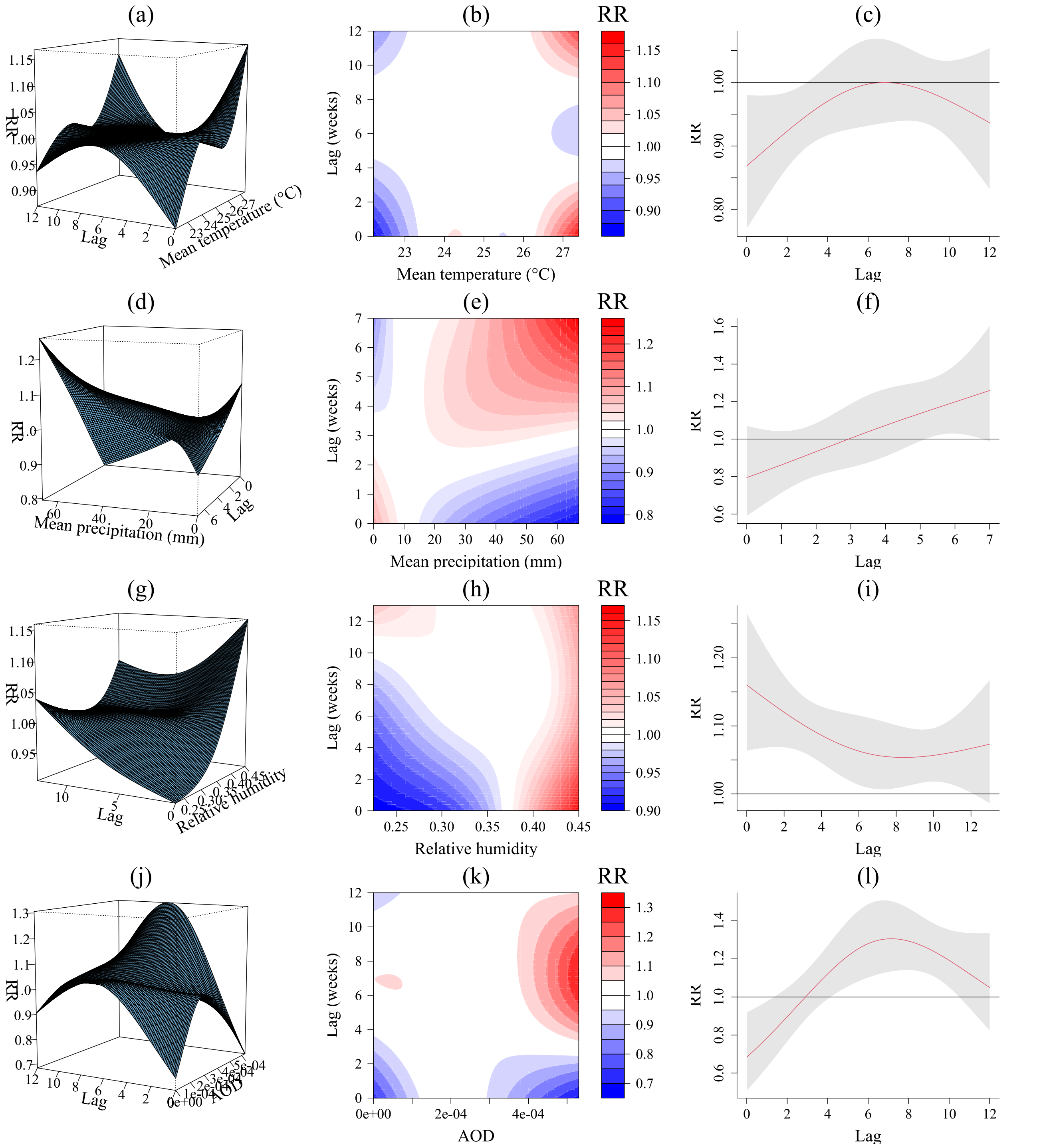

Figure S8. Exposure–lag–response associations between climatic and air-quality predictors and the relative risk (RR) of weekly respiratory hospital discharges in subregion RMS1, estimated from the optimal distributed lag nonlinear mixed model (zero-inflated negative binomial with subregional random effects). Rows correspond to temperature (T), precipitation (P), relative humidity (RH), and aerosol optical depth (AOD). Panels (a,d,g,j) show three-dimensional surfaces; (b,e,h,k) contour plots; and (c,f,i,l) lag–response curves (lag in weeks) evaluated at representative exposure levels (observed maxima for T and P, and observed minima for RH and AOD). Colors indicate the magnitude of RR (red: RR > 1; blue: RR < 1). Predictions are centered at the subregional mean exposure.

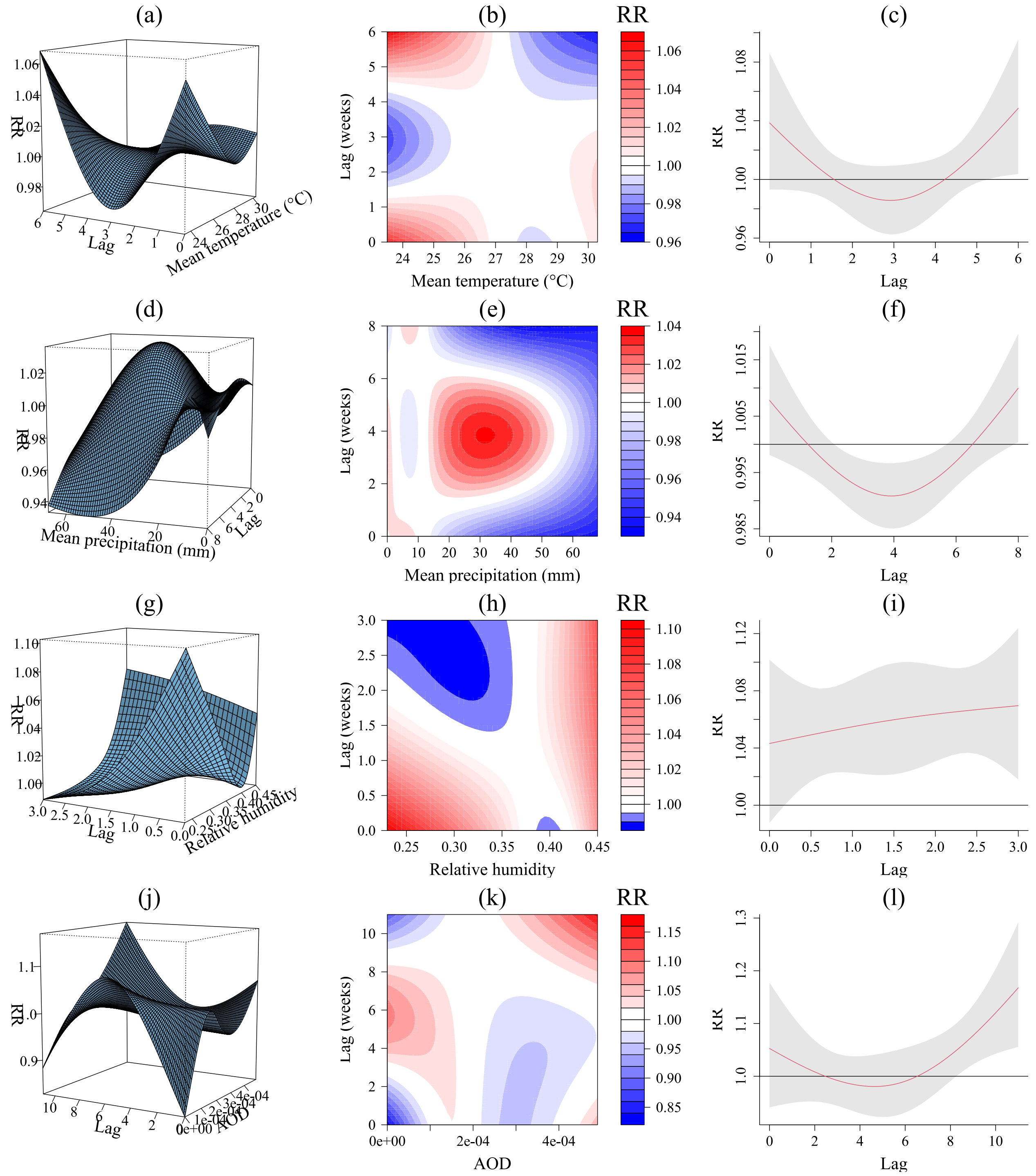

Figure S9. Exposure–lag–response associations between climatic and air-quality predictors and the relative risk (RR) of weekly respiratory hospital discharges in subregion RN1 , estimated from the optimal distributed lag nonlinear mixed model (negative binomial with subregional random effects). Rows correspond to temperature (T), precipitation (P), relative humidity (RH), and aerosol optical depth (AOD). Panels (a,d,g,j) show three-dimensional surfaces; (b,e,h,k) contour plots; and (c,f,i,l) lag–response curves (lag in weeks) evaluated at representative exposure levels ($T = 24.6$°C, $P = 7$ mm, and observed maxima for RH and AOD). Colors indicate the magnitude of RR (red: RR > 1; blue: RR < 1). Predictions are centered at the subregional mean exposure.

Table S4. Significant (5%) exposure–lag ranges and the corresponding types of significant relative risk (RR)—Low (RR $<$ 1) and High (RR $>$ 1)—for each climatic predictor (temperature (T), precipitation (P), relative humidity (RH), and aerosol optical depth (AOD)) from the best-fitted model across climatic subregions (RA, RMS, and RN).

Region	Variable	Exposure range		Lag range		RR type
		Minimum	Maximum	Minimum	Maximum	
RA	Temperature	16.0	16.9	0	3	Low
		20.1	21.0	1	2	High
	Precipitation	13	29	4	6	Low
		38	60	3	6	High
	Relative humidity	0.230	0.370	7	11	Low
		0.375	0.450	1	11	High
	AOD	0.00000	0.00006	0	11	Low
		0.00007	0.00008	4	7	Low
		0.00041	0.00057	0	5	High
RMS	Temperature	22.2	22.8	0	3	Low
		26.8	27.4	0	12	High
	Precipitation	54	67	4	7	High
	Relative humidity	0.225	0.370	0	4	Low
		0.375	0.450	0	7	High
	AOD	0.00000	0.00008	0	3	Low
		0.00041	0.00043	0	7	High
RN	Temperature	24.4	27.1	5	6	High
		27.2	28.9	4	6	Low
	Precipitation	6	7	2	6	Low
		30	45	2	5	High
	Relative humidity	0.400	0.450	0	2	High
	AOD	0.00020	0.00024	0	9	Low
		0.00042	0.00049	8	11	High

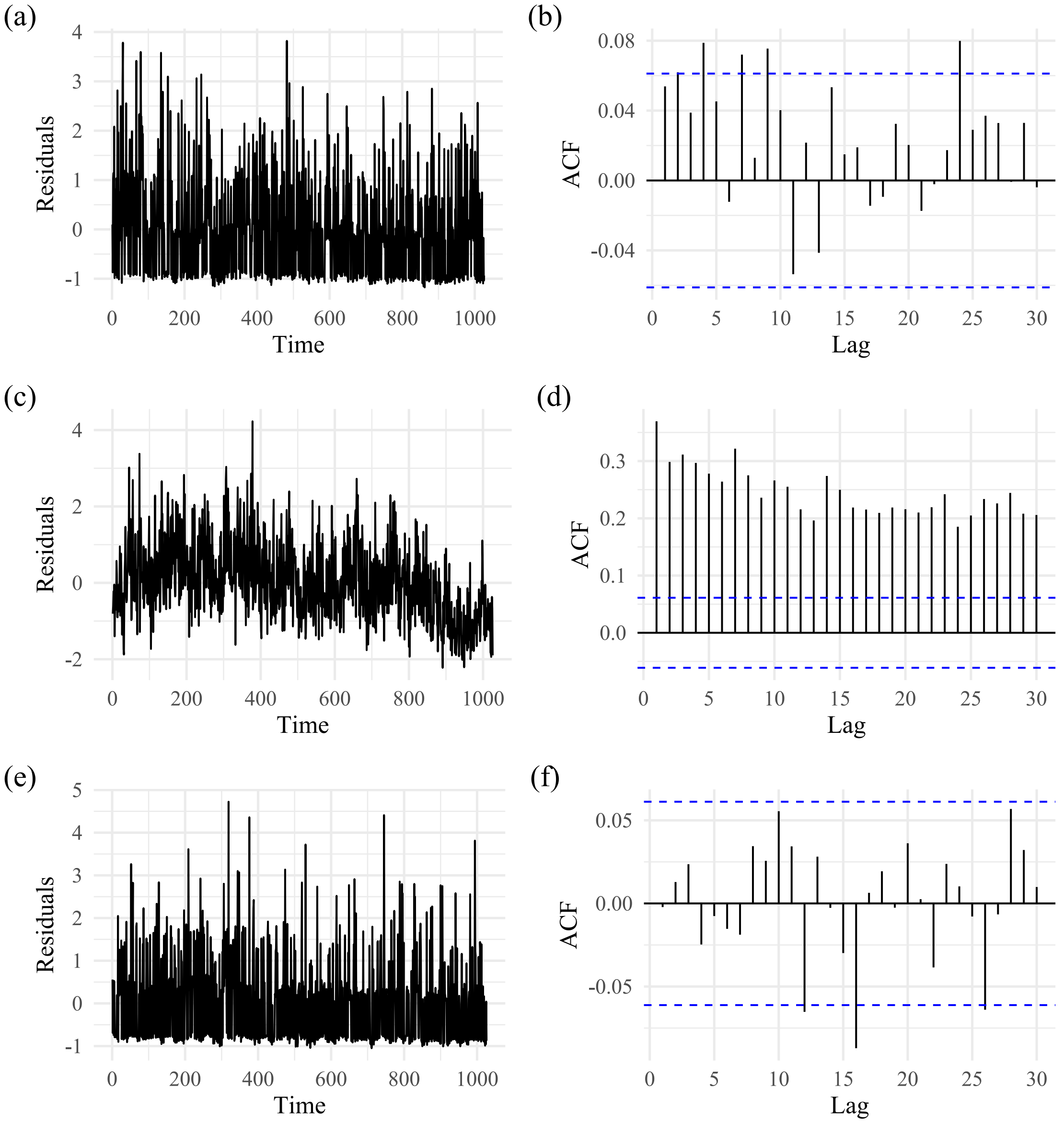

Figure S10. Diagnostic plots of Pearson residuals from the optimal distributed lag nonlinear mixed model (DLNM–GLMM) fitted with a negative binomial distribution. Panels (a)–(b), (c)–(d), and (e)–(f) correspond to subregions PC1, PC2, and PC3 of the Central Pacific Region, respectively. For each subregion, the left panel shows the Pearson residual time series and the right panel its autocorrelation function (ACF). Dashed horizontal lines indicate approximate 95% confidence limits for the null hypothesis of no serial correlation.

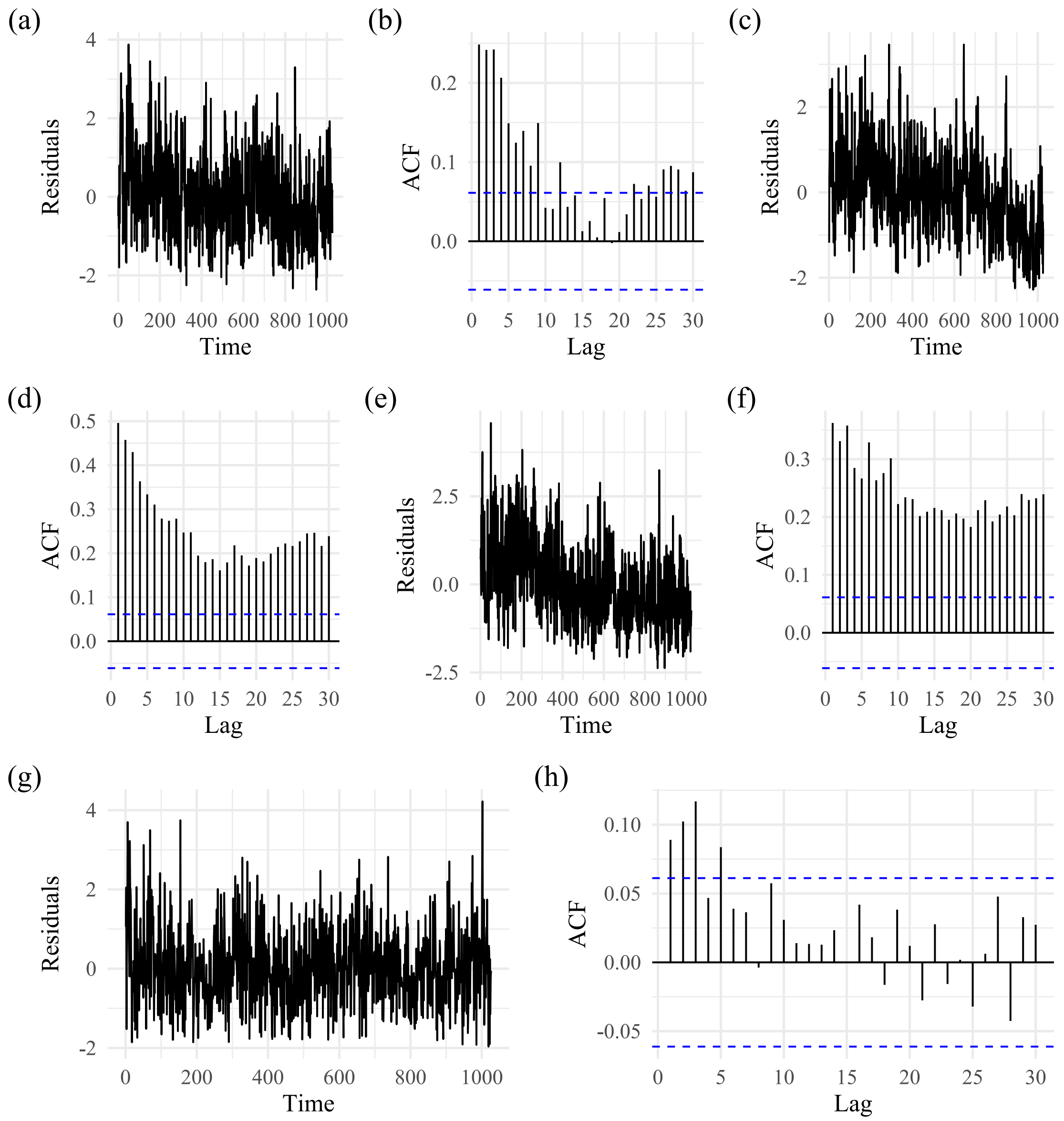

Figure S11. Diagnostic plots of Pearson residuals from the optimal distributed lag nonlinear mixed model (DLNM–GLMM) fitted with a negative binomial distribution. Panels (a)–(b), (c)–(d), (e)–(f), and (g)–(h) correspond to subregions PN1, PN2, PN3, and PN4 of the North Pacific Region, respectively. For each subregion, the left panel shows the Pearson residual time series and the right panel its autocorrelation function (ACF). Dashed horizontal lines indicate approximate 95% confidence limits for the null hypothesis of no serial correlation.

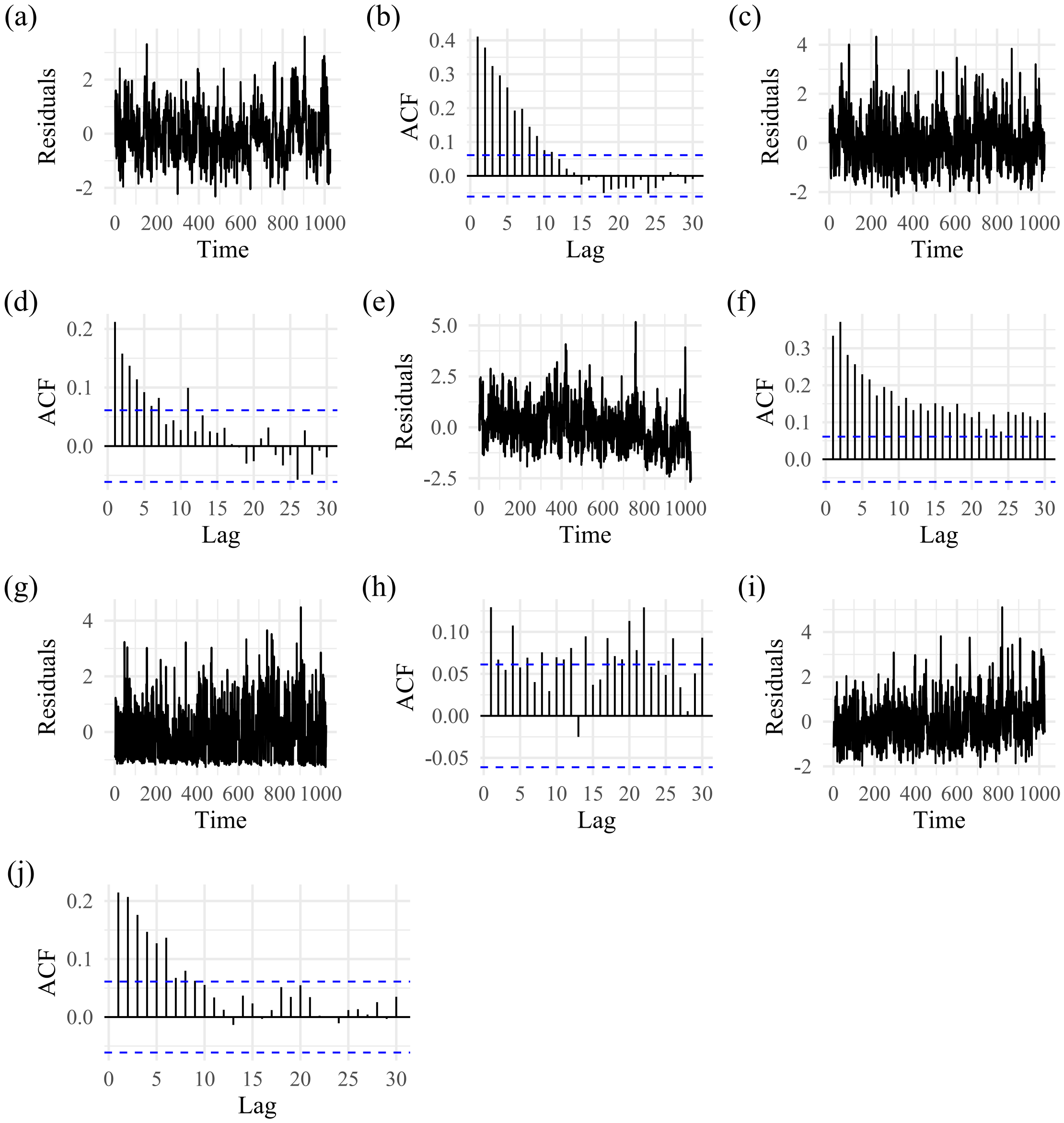

Figure S12. Diagnostic plots of Pearson residuals from the optimal distributed lag nonlinear mixed model (DLNM–GLMM) fitted with a negative binomial distribution. Panels (a)–(b), (c)–(d), (e)–(f), (g)–(h), and (i)–(j) correspond to subregions PS1, PS2, PS3, PS4, and PS5 of the South Pacific Region, respectively. For each subregion, the left panel shows the Pearson residual time series and the right panel its autocorrelation function (ACF). Dashed horizontal lines indicate approximate 95% confidence limits for the null hypothesis of no serial correlation.

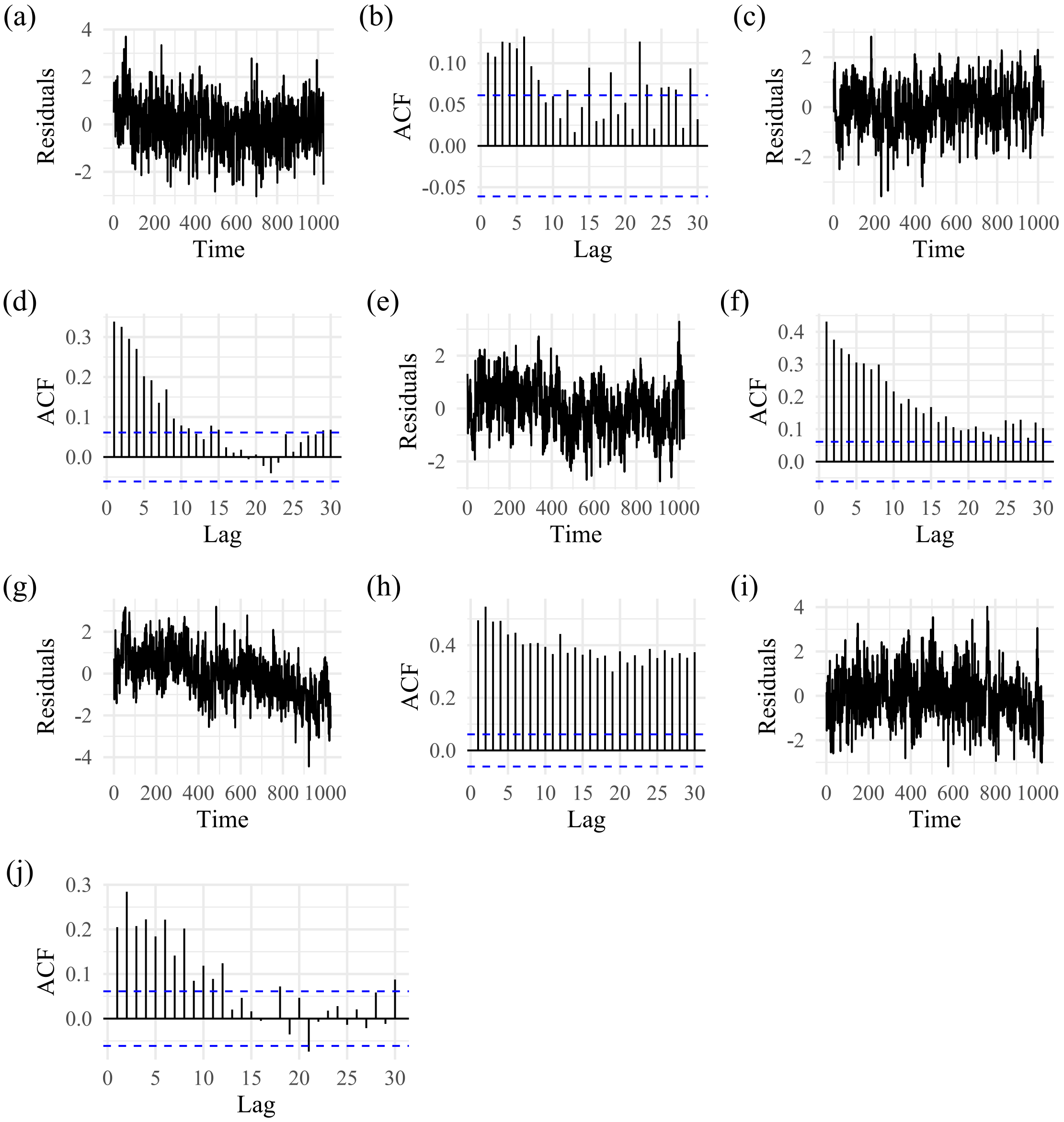

Figure S13. Diagnostic plots of Pearson residuals from the optimal distributed lag nonlinear mixed model (DLNM–GLMM) fitted with a zero-inflated negative binomial distribution. Panels (a)–(b), (c)–(d), (e)–(f), (g)–(h), and (i)–(j) correspond to subregions RA1, RA2, RA3, RA4, and RA5 of the Atlantic Region (RA), respectively. For each subregion, the left panel shows the Pearson residual time series and the right panel its autocorrelation function (ACF). Dashed horizontal lines indicate approximate 95% confidence limits for the null hypothesis of no serial correlation.

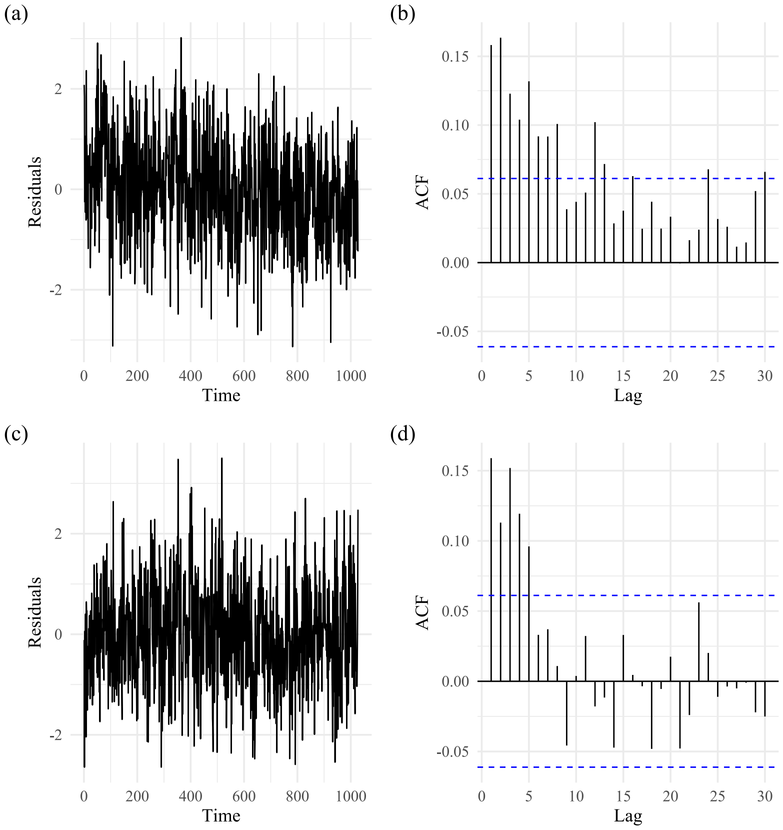

Figure S14. Diagnostic plots of Pearson residuals from the optimal distributed lag nonlinear mixed model (DLNM–GLMM) fitted with a zero-inflated negative binomial distribution. Panels (a)–(b) and (c)–(d) correspond to subregions RMS1 and RMS2 of the Southern Mountain Region, respectively. For each subregion, the left panel shows the Pearson residual time series and the right panel its autocorrelation function (ACF). Dashed horizontal lines indicate approximate 95% confidence limits for the null hypothesis of no serial correlation.

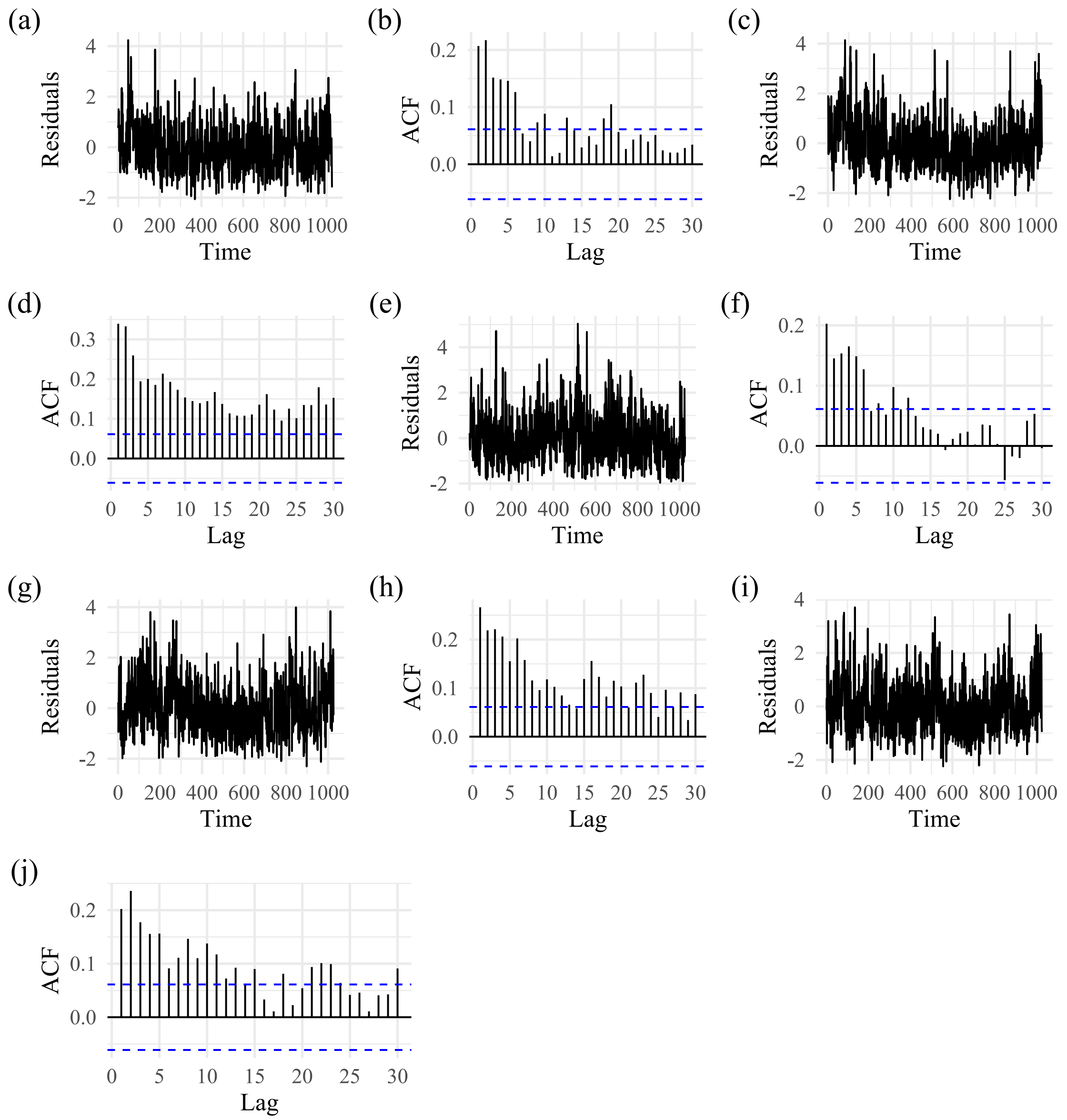

Figure S15. Diagnostic plots of Pearson residuals from the optimal distributed lag nonlinear mixed model (DLNM–GLMM) fitted with a negative binomial distribution. Panels (a)–(b), (c)–(d), (e)–(f), (g)–(h), and (i)–(j) correspond to subregions RN1, RN2, RN3, RN4, and RN5 of the Northern Region (RN), respectively. For each subregion, the left panel shows the Pearson residual time series and the right panel its autocorrelation function (ACF). Dashed horizontal lines indicate approximate 95% confidence limits for the null hypothesis of no serial correlation. These diagnostics assess residual independence and overall model adequacy.
